# Multi-feature fusion for grassland information extraction and temporal change monitoring

**DOI:** 10.1371/journal.pone.0347821

**Published:** 2026-11-06

**Authors:** Xiyuan Liu, Jie Chen, Zijin Liu, Xiaohua Zhu, Shigao Jiang, Wanli Kang

**Affiliations:** 1 Shaoyang Grassland Resources Protection Center, Shaoyang, Hunan, China; 2 College of Civil Engineering, Xiangtan University, Xiangtan, Hunan, China; Universidade Federal de Uberlandia, BRAZIL

## Abstract

Grasslands play a vital role in maintaining ecological security and supporting sustainable development, yet accurate extraction and dynamic monitoring remain challenging in complex mountainous regions. This study takes Chengbu Miao Autonomous County, Hunan Province, as the study area and develops a multi-feature fusion framework on the Google Earth Engine platform by integrating optical imagery, Sentinel-1 SAR data, vegetation indices, texture features, topographic factors, and time-series characteristics. A Random Forest model was employed for high-accuracy grassland extraction, while multi-temporal remote sensing observations from selected cloud-free years between 2000 and 2025 were used to analyze long-term spatial distribution and temporal dynamics. Fractional vegetation cover (FVC) was introduced to assess grassland ecological conditions, and Nanshan Pasture was selected to investigate the impacts of topography, human activities, and climatic factors on grassland variation. The results show that multi-feature fusion significantly improves classification accuracy, achieving an overall accuracy (OA) of 93.79% and a Kappa coefficient of 0.7165 in the optimal feature combination scheme, with optical and SAR data exhibiting strong complementarity under complex terrain conditions. Grassland area in Chengbu County remained relatively stable from 2000 to 2025, with annual variations ranging from 47.32 to 52.15 km² and no significant long-term change trend. The mean FVC exhibited relatively stable interannual fluctuations, with values generally ranging from 0.59 to 0.72 during most years. However, spatial analysis of FVC trends revealed considerable heterogeneity, with significant decreasing trends observed in localized areas, particularly in the southwestern part of Nanshan Pasture, indicating potential vegetation degradation risks. In Nanshan Pasture, FVC showed a significant negative correlation with DEM (r = −0.2839) and precipitation (r = −0.589), while correlations with human activities and land surface temperature were relatively weak. Overall, grassland dynamics in the study area may reflect the combined effects of environmental conditions and human activities, although the relationships identified in this study mainly represent statistical associations rather than causal mechanisms. The proposed framework provides an effective methodological reference for grassland monitoring, ecological assessment, and sustainable management in subtropical mountainous grassland environments.

## 1. Introduction

Grasslands are an essential component of terrestrial ecosystems, playing an irreplaceable role in maintaining ecological security patterns, regulating regional climate, conserving soil and water, and sustaining biodiversity [1,2]. As an important ecological transitional type linking forests and croplands, grasslands play a crucial regulatory role in carbon cycling, energy exchange, and material transport processes [3,4]. Meanwhile, grassland resources constitute the material foundation for the development of animal husbandry, and their spatial distribution and quality conditions are directly linked to regional ecological stability and sustainable socio-economic development [5]. However, under the combined influences of climate change and human activities, issues such as grassland degradation, fragmentation, and changes in utilization patterns have become increasingly prominent. This underscores the urgent need for accurate and continuous monitoring of grassland spatial patterns and their temporal dynamics using scientific approaches [6,7].

With the deepening understanding of grassland ecological functions and their dynamic changes, achieving high-accuracy, long-term time-series monitoring of grassland information has become a key research topic in the fields of ecology and resource management [8]. Traditional field survey methods are constrained by high labor costs, limited spatial coverage, and insufficient temporal efficiency, making it difficult to meet the practical demands of large-scale and continuous monitoring. In contrast, remote sensing technology, with its advantages of synoptic coverage, periodic observation, and repeatability, provides an effective means for identifying grassland spatial distribution and monitoring their dynamic changes [9,10]. The development of cloud-based geospatial platforms, such as Google Earth Engine (GEE), has further facilitated the integration of multi-source remote sensing and environmental datasets for large-scale ecological monitoring and assessment [11,12]. In regions such as Hunan Province, characterized by complex terrain and diverse ecosystems, the monitoring and management of grassland resources have received increasing attention from both governmental authorities and the scientific community. Relevant provincial policies emphasize the establishment of an integrated air–space–ground grassland survey and dynamic monitoring system, along with standardized grassland resource database construction and annual monitoring and evaluation mechanisms, aiming to clarify baseline resource conditions and support ecological protection and utilization decisions. In scientific practice, the integration of multi-source remote sensing data has been widely applied to estimate key ecological indicators, such as biomass, in typical grasslands of Hunan Province, demonstrating significant potential for improving accuracy. However, existing studies have largely focused on data-source synergy or the application of individual features, and there remains a lack of systematic multi-feature fusion approaches tailored to grassland heterogeneity, as well as long-term time-series change models. Consequently, the complex characteristics of grasslands in terms of spatial patterns, temporal dynamics, and ecological processes have yet to be comprehensively characterized [13,14].

Overall, although notable progress has been made in remote sensing-based grassland monitoring, it still faces numerous challenges under complex surface conditions. This is particularly evident in regions such as Hunan Province, where hilly and mountainous terrains dominate, land cover types are highly fragmented, and grassland types are diverse [15]. In these subtropical mountainous grassland ecosystems, complex terrain conditions, including elevation gradients, slope variations, and illumination differences, introduce substantial spatial variability in vegetation signals. Meanwhile, fragmented grassland distribution and frequent transitions among grassland, forest, cropland, and shrub ecosystems increase spectral confusion, making accurate grassland identification challenging. Therefore, relying on a single feature or data source is often insufficient for stable grassland information extraction [16]. Meanwhile, existing studies are predominantly based on single-date or short-term analyses, with insufficient attention paid to the long-term evolution processes and temporal dynamics of grasslands. For mountainous grassland ecosystems in Hunan Province, where vegetation dynamics are jointly influenced by topographic conditions, climatic variability, and human activities, short-term observations may not adequately characterize long-term ecological changes and their driving mechanisms. This limitation constrains in-depth investigations into grassland dynamic monitoring and the underlying mechanisms of change [17]. Therefore, it is imperative to develop an integrated methodological framework that incorporates multi-source remote sensing features while jointly accounting for the spectral, structural, and temporal characteristics of grasslands. Such an approach is expected to improve the accuracy of grassland information extraction and enable refined monitoring of grassland spatiotemporal dynamics at the regional scale, thereby providing reliable data support for grassland resource conservation and scientific management in Hunan Province.

Building upon the aforementioned challenges, this study develops an integrated framework for grassland information extraction and temporal change monitoring in complex mountainous environments. Unlike previous studies that mainly combine conventional remote sensing datasets with machine learning classifiers for land-cover mapping, this study proposes a comprehensive workflow that extends beyond classification accuracy evaluation by integrating multi-source feature fusion, feature importance-based model interpretation, long-term grassland dynamic monitoring, and environmental driver analysis. Specifically, the Google Earth Engine (GEE) platform was employed for multi-source remote sensing data acquisition and preprocessing, including optical imagery, SAR data, and environmental auxiliary datasets. Based on these datasets, a hierarchical multi-dimensional feature system was constructed by integrating optical spectral information, vegetation indices, SAR backscattering characteristics, texture features, topographic variables, and temporal characteristics, thereby characterizing the spectral, structural, and environmental heterogeneity of mountainous grasslands. The Random Forest (RF) model was implemented in MATLAB for grassland classification, feature importance analysis, and accuracy assessment. In addition to classification, RF was further utilized to quantify the contribution of different features, providing insights into the decision-making process of the model and improving the interpretability of grassland extraction. Furthermore, the extracted grassland information was combined with fractional vegetation cover (FVC) and environmental factors, including topographic conditions, human activity intensity, and climatic variables, to investigate the mechanisms driving grassland changes from multiple perspectives. Therefore, this study establishes a comprehensive workflow integrating data acquisition, multi-source feature fusion, model interpretation, long-term monitoring, and ecological assessment, providing a methodological framework and practical reference for grassland resource management and ecological conservation in subtropical mountainous grassland environments.

To achieve the above goals, this study focuses on three specific objectives: (1) evaluating the effectiveness of multi-source feature fusion for grassland classification under complex mountainous environments; (2) establishing a long-term monitoring framework to characterize grassland spatial and temporal dynamics; and (3) exploring the potential relationships between grassland ecological changes and environmental driving factors. The main scientific contributions of this study are summarized as follows:

(1) A systematic multi-feature evaluation strategy was developed for subtropical mountainous grassland classification by constructing and comparing 20 feature combination schemes. This approach quantitatively reveals the complementary roles of optical spectral information, vegetation indices, SAR-derived structural characteristics, texture features, and topographic variables in grassland discrimination.
(2) A long-term grassland monitoring framework was established for a typical mountainous grassland region in Hunan Province, China, where grassland ecosystems are characterized by high fragmentation, complex terrain, and strong spectral heterogeneity. The framework integrates multi-temporal remote sensing observations to investigate long-term variations in grassland distribution and vegetation conditions.
(3) Beyond land-cover classification, this study integrates fractional vegetation cover (FVC) analysis with topographic, climatic, and anthropogenic factors to explore the potential mechanisms driving grassland ecological changes, providing a more comprehensive understanding of grassland dynamics under combined environmental and human influences.

## 2. Study area and data sources

### 2.1. Overview of the study area

Chengbu Miao Autonomous County, administered under Shaoyang City, is located in the western border region of Hunan Province, central-southern China (Fig 1). It lies approximately between 109°58′–110°37′ E and 25°58′–26°42′ N. The elevation ranges from 400 to 2023 m, with an average elevation of nearly 700 m, and a total area of about 2647.07 km². The region is characterized by a mid-subtropical mountainous climate. Topographically, elevations are higher in the southeast and northwest, while the central and northeastern parts are relatively lower, exhibiting highly pronounced terrain variability.

**Fig 1:**
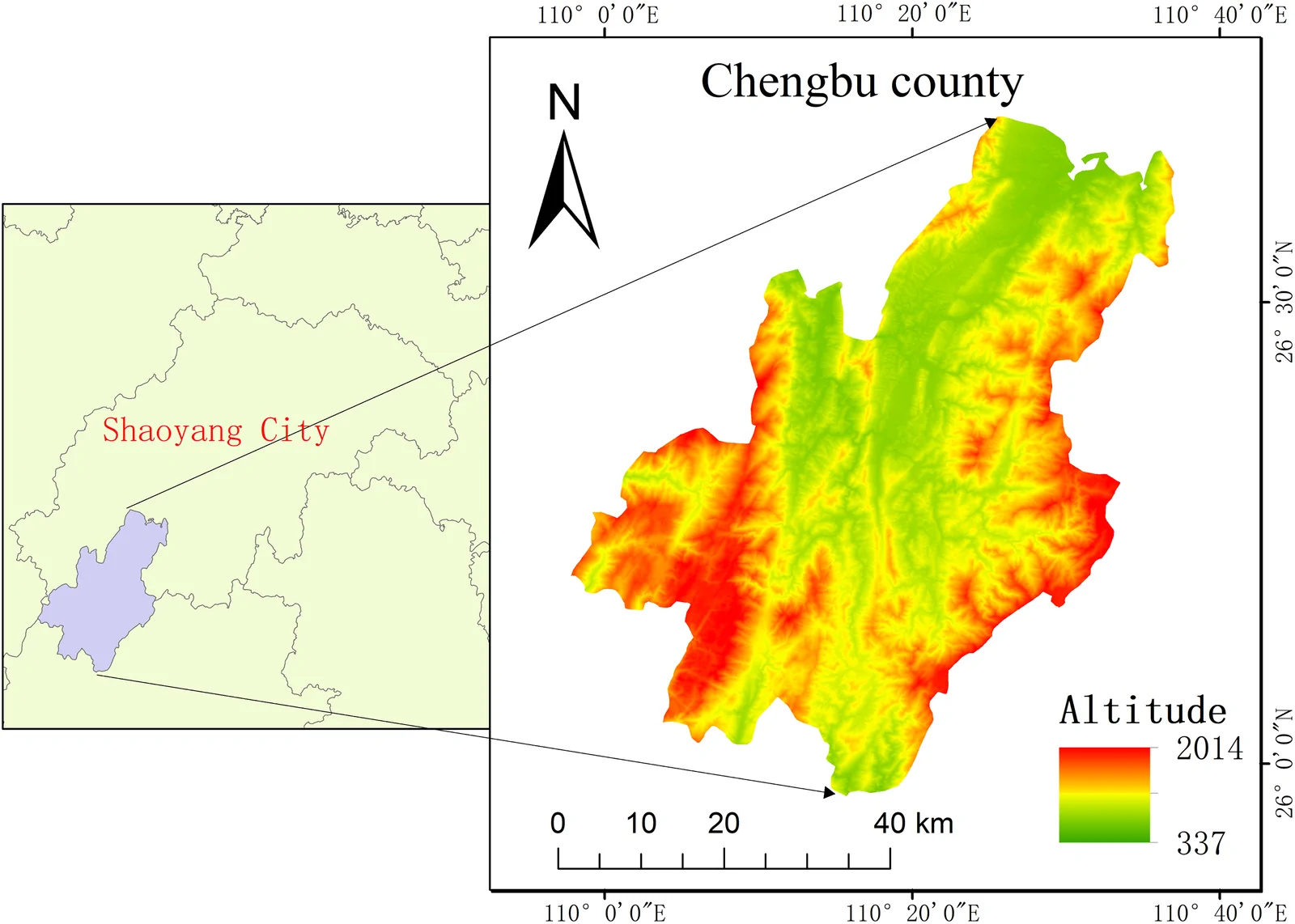
Overview of the study area.

The mid-subtropical mountainous climate of Chengbu provides abundant heat and moisture, endowing vegetation with high productivity potential. Meanwhile, the mid- to high-elevation zones constrain the growth of tall trees, thereby creating favorable ecological niches for herbaceous plants. In the southwestern part of Chengbu, there exists a large high-elevation plateau—Nanshan. Characterized by a flat and open surface, good drainage conditions, sufficient solar radiation, and the accumulation of soil organic matter, Nanshan offers ideal terrain for the formation of concentrated, high-quality grasslands. Grasslands in Chengbu, particularly the Nanshan Pasture, are the result of the coupled effects of four key factors: high-elevation plateau topography, cool and humid climatic conditions, fertile meadow soils, and localized hydrological conditions.

### 2.2. Data sources and preprocessing

#### 2.2.1. Remote sensing data.

Optical remote sensing imagery, vegetation indices, and texture features were derived from Landsat data with a spatial resolution of 30 m, covering the study area from March to September during 2000–2025 on the Google Earth Engine (GEE) platform. The datasets include Landsat 5 MSS/TM and Landsat 8/9 OLI/TIRS (Datasets: LANDSAT/LT05/C02/T1_L2, LANDSAT/LC08/C02/T1_L2, and LANDSAT/LC09/C02/T1_L2, all of which have been preprocessed with atmospheric correction, radiometric calibration, and geometric correction.), which have been preprocessed with atmospheric correction, radiometric calibration, and geometric correction. Additional preprocessing steps, including cloud masking and clipping to the study area, were further applied. Topographic features were obtained from the Shuttle Radar Topography Mission (SRTM) dataset (Datasets: USGS/SRTMGL1_003) with a spatial resolution of 30 m on the GEE platform, followed by clipping to the study area. SAR imagery was acquired from the Sentinel-1 dual-polarization C-band Synthetic Aperture Radar (SAR) dataset (Datasets: COPERNICUS/S1_GRD) with a spatial resolution of 10 m, covering the period from March to September during 2015–2025. The Sentinel-1 images were preprocessed in the Google Earth Engine (GEE) platform, including conversion from decibel (dB) values to the linear scale, terrain correction, speckle noise reduction, and spatial clipping. Terrain correction was performed using the SRTM DEM dataset to reduce the influence of mountainous terrain on radar backscattering responses. Subsequently, Refined Lee filtering was applied in the linear domain to suppress speckle noise while preserving spatial texture information. Specifically, a local statistical filtering strategy with 3 × 3 and 7 × 7 moving windows was adopted to estimate local mean and variance information. After filtering, the data were converted back to the dB scale for subsequent feature extraction. Finally, SAR-derived features, including VV, VH, VH/VV ratio, RVI, and DpRVI, were generated. To ensure spatial consistency among multi-source datasets, all datasets used in this study were referenced to the WGS 1984 coordinate datum. All remote sensing images and auxiliary datasets were spatially matched, clipped to the study area, and resampled to a unified spatial resolution of 30 m using the nearest-neighbor interpolation method before further analysis. Sentinel −2 imagery was used only to assist visual interpretation and reference sample delineation, rather than as input features for Random Forest classification. Landsat data were selected because they provide consistent 30 m observations covering the full 2000–2025 period.

#### 2.2.2. Sample data.

Field survey data collected during 2021–2024 were used as the primary reference information for sample construction. By integrating field survey data with high-resolution imagery from Google Earth and Sentinel-2 imagery with a spatial resolution of 10 m, a visual interpretation approach was employed to delineate land cover types throughout the study area. Six land cover categories were identified, including built-up land, cropland, water bodies, forest, bare land, and grassland, and a reference sample dataset was subsequently established. The visual interpretation was conducted by a single experienced interpreter following consistent interpretation criteria established from field observations and multi-source reference imagery. During the interpretation process, land-cover identification was supported by spectral characteristics, vegetation distribution patterns, spatial context, and field survey information to reduce potential subjective uncertainty.

Although the field survey samples were collected during 2021–2024, they were mainly used to characterize the spectral and ecological properties of the major land-cover categories in the study area. The application of these samples to historical Landsat images from 2000 onward assumes that the fundamental characteristics of relatively stable land-cover types, particularly grassland ecosystems, have remained comparable over time. This assumption is reasonable for the mountainous pasture-dominated landscape of Chengbu County, where large-scale land-cover transitions are relatively limited compared with highly urbanized or intensively cultivated regions. However, potential changes caused by ecological restoration programs, agricultural activities, and land management practices may introduce uncertainties into retrospective land-cover classification. These uncertainties are further discussed in the Limitations and Future Perspectives section.

During the sample construction process, the spatial heterogeneity of the study area was considered. Pixel-based reference samples were manually delineated across the entire study area to cover different land cover types, spatial environments, and topographic conditions, ensuring the representativeness of the sample dataset. The generated reference dataset contained a total of 2,875,111 labeled pixels, including 8,996 built-up land pixels, 234,044 cropland pixels, 12,900 water body pixels, 2,471,387 forest pixels, 2,813 bare land pixels, and 64,069 grassland pixels. Due to the substantial differences in spatial extent among land cover categories in the mountainous study area, a non-balanced sampling strategy was adopted to preserve the actual distribution characteristics of surface types rather than artificially equalizing the number of samples among classes. Considering the large number of generated labeled pixels and the requirement for reliable independent accuracy assessment, the reference dataset was randomly divided into a training set (65%) and a validation set (35%). However, due to the spatial continuity of remote sensing data, adjacent pixels may share similar spectral, topographic, and textural characteristics, which may introduce potential spatial autocorrelation between training and validation samples. This limitation was considered in the interpretation of accuracy assessment results and is further discussed in the Limitations and Future Perspectives section. This ratio was selected to provide sufficient samples for Random Forest model training while maintaining an independent dataset for accuracy evaluation. The random partitioning procedure ensured that both datasets retained similar class proportions and spatial coverage, thereby reducing sampling bias and improving the reliability of model evaluation. Finally, 1,868,822 pixels were used for model training and 1,006,289 pixels were used for validation.

#### 2.2.3. Climate data.

Climate data were obtained from the CHIRPS daily precipitation dataset and the MODIS land surface temperature (LST) product available on the Google Earth Engine (GEE) platform, with LST used as a proxy for air temperature. The spatial resolution of the precipitation data is approximately 5 km, while the LST data have a spatial resolution of 1 km (Precipitation dataset: UCSB-CHG/CHIRPS/DAILY; LST dataset: MODIS/061/MOD11A2).

#### 2.2.4. Auxiliary data.

Grassland survey data: Field survey data collected during 2021–2024 were used as reference information for land-cover sample construction. A total of 102 field survey points were collected, including 19 points in 2021, 29 points in 2022, 27 points in 2023, and 27 points in 2024. The field observations mainly covered grassland and other major land-cover categories within Chengbu County, Hunan Province, and were distributed across representative vegetation conditions and terrain environments.The geographic coordinates of each survey point were recorded using GNSS positioning equipment and were spatially matched with corresponding remote sensing pixels. These field observations were combined with high-resolution remote sensing imagery and Sentinel-2 imagery (10 m spatial resolution) for visual interpretation and reference sample generation. During the preprocessing procedure, field points were first checked according to their land-cover consistency, and representative samples were subsequently selected to construct the training dataset for Random Forest classification.The field survey data were mainly used for land-cover sample construction and were not used as independent validation samples for accuracy assessment. The surveyed locations provided reliable reference information for characterizing grassland spectral and ecological properties, supporting subsequent multi-source feature analysis and classification experiments.

Human activity intensity data: in this study, a proxy dataset representing human activity intensity was constructed based on 0.5 m high-resolution remote sensing imagery acquired in 2026. Human activity-related features, including residential areas and major roads, were manually interpreted from the imagery. During the construction of the dataset, both the spatial distribution and area extent of residential areas were considered to characterize the intensity of anthropogenic disturbance. Subsequently, Euclidean distance analysis was performed on residential areas and major roads, followed by spatial reclassification, raster calculation, and grading in ArcGIS to generate a 30 m resolution human activity intensity proxy dataset. This dataset was subsequently used to evaluate the potential influence of anthropogenic activities on grassland FVC variation.

## 3. Methodology

### 3.1. Overall technical workflow

The technical workflow for grassland classification and extraction is illustrated in Fig 2, consisting of two main modules: data acquisition and data processing. Specifically, the workflow includes seven key steps: data preprocessing, feature extraction, sample dataset construction, Random Forest classification, accuracy assessment and comparative analysis, time-series analysis, and correlation analysis. In the data preprocessing stage, optical imagery and texture features are subjected to cloud removal, median compositing, and clipping, while SAR imagery undergoes terrain correction, Refined Lee filtering, and clipping. Feature extraction is conducted based on Landsat-5/8/9 optical imagery, Sentinel-1 SAR data, and DEM, from which optical indices, radar indices, slope, and aspect are derived. During the sample dataset construction stage, sampling regions are delineated according to land cover types, and the samples are divided into training and validation sets. Subsequently, the Random Forest algorithm is employed to perform land cover classification using the training samples and the extracted features. The classification results are then evaluated through accuracy assessment and comparative analysis to verify the effectiveness of the proposed method. Finally, the trained Random Forest model is applied to classify data from 2015 to 2025, generating annual land cover maps and grassland-specific extraction results. Based on these outputs, time-series change characteristics and correlation analyses are further conducted.

**Fig 2:**
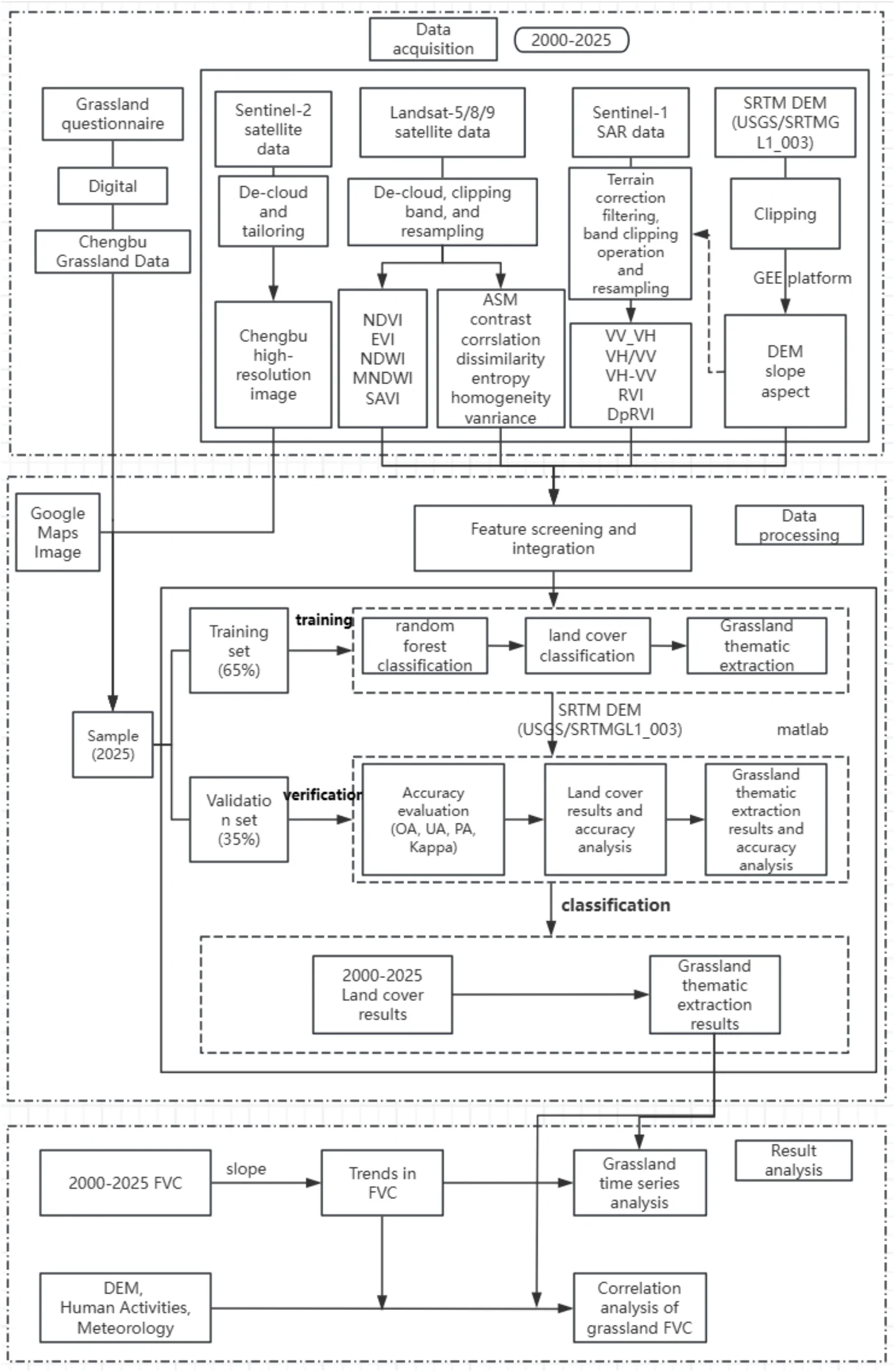
Research workflow.

### 3.2. Feature extraction and construction

In this study, five categories of features were extracted, including original spectral features, vegetation index features, SAR features, topographic features, and texture features, as summarized in Table 1. The original spectral features consist of Landsat bands B1–B7. For vegetation index features, the enhanced vegetation index (EVI), normalized difference vegetation index (NDVI), normalized difference water index (NDWI), modified normalized difference water index (MNDWI), and soil-adjusted vegetation index (SAVI) were selected. SAR features were derived from Sentinel-1 dual-polarization data, including VV and VH backscatter coefficients. Additional SAR-derived features include the ratio vegetation index (RVI), VH/VV ratio, VH–VV difference in dB, and the dual-polarimetric radar vegetation index (DpRVI). Topographic features include the digital elevation model (DEM), slope, and aspect. Texture features were calculated based on gray-level co-occurrence matrix (GLCM) metrics, including angular second moment (ASM), contrast, correlation, dissimilarity, entropy, homogeneity, and variance.

**Table 1:** Description of features.

Feature Type	Spectral Band Information and Formulas	Note
Original Spectral Features	B1, B2, B3, B4, B5, B6, B7	–
Optical Index Features	$NDVI=\frac{NIR-RED}{NIR+RED}$	RED: red band; NIR: near-infrared band
	$EVI=\text{2}.\text{5}\times \frac{NIR-RED}{NIR-\text{6}\times RED-\text{7}.\text{5}\times BLUE+\text{1}}$	BLUE: blue band
	$NDWI=\frac{GREEN-NIR}{GREEN+NIR}$	GREEN: green band
	$MNDWI=\frac{GREEN-SWIR}{GREEN+SWIR}$	SWIR: shortwave infrared band
	$SAVI=\frac{NIR-RED}{NIR+RED+L}\times (\text{1}+L)$	L: soil adjustment factor (set to 0.5 in this study)
SAR Features	VV, VH	VV: co-polarization (vertical transmit, vertical receive); VH: cross-polarization (vertical transmit, horizontal receive)
	$R=\frac{\sigma^{0}\_VH}{\sigma^{0}\_VV}$	σ°_VH: backscatter coefficient (VH polarization)
	$RVI=\frac{\text{4}\times \sigma^{0}\_VV}{\sigma^{0}\_VV\;+\;\sigma^{0}\_VH}$	σ°_VV: backscatter coefficient (VV polarization)
SAR Features	$DpRVI=\left(\text{1}-\frac{\alpha }{\alpha +\text{1}}\right)\times \text{2}\;,where\;\alpha =\frac{\sigma^{0}\_VV\;}{\sigma^{0}\_VH}$	σ°_VH_dB: backscatter coefficient in decibel (dB) scale for vertical transmit–horizontal receive polarization
	$\Delta_{VH-VV}=\sigma^{0}\_VH\_dB-\sigma^{0}\_VV\_dB$	σ°_VV_dB: backscatter coefficient in decibel (dB) scale for vertical transmit–vertical receive polarization
Topographic Features	DEM, slope, aspect	DEM: Digital Elevation Model; slope: terrain slope; aspect: terrain aspect
Texture Features (GLCM-based)	ASM, contrast, correlation, dissimilarity, entropy, homogeneity, variance	ASM: Angular Second Moment

### 3.3. Feature importance analysis and feature combination scheme

Feature importance analysis provides insights into the contribution of different input variables within machine learning models, helping to evaluate the relative influence of features, identify redundant information, and support feature combination optimization. In this study, the out-of-bag (OOB) permutation importance method of the Random Forest (RF) model was employed to quantify the relative contribution of each feature to model prediction performance. The obtained importance scores were used to evaluate the contribution of different remote sensing features and guide the construction of feature combination schemes. Rather than representing direct causal relationships, the feature importance values reflect the contribution of individual variables to the model classification process [18,19].

To investigate the contribution and complementary effects of different remote sensing information sources, all input features were categorized into five groups according to their physical characteristics and ecological meanings: (A) SAR features, (B) optical vegetation indices, (C) texture features, (D) topographic variables, and (E) original spectral bands. Based on these feature groups, 20 feature combination schemes were constructed (Table 2). These schemes followed a progressive fusion strategy, including single-feature groups, multi-feature combinations, and comprehensive multi-source fusion. The objective was to evaluate the contribution of different information sources and determine whether the integration of complementary remote sensing features could improve grassland classification performance. To ensure comparability among experiments, all feature combination schemes were evaluated using the same Random Forest classifier and parameter settings. Therefore, differences in classification accuracy among schemes mainly reflected the contribution of feature selection and fusion strategies.

**Table 2:** Feature combination schemes.

Scheme	Feature Combination	Scheme	Feature Combination
1	A	11	A, B, D
2	B	12	A, B, E
3	C	13	A, C, D
4	D	14	A, C, E
5	E	15	A, D, E
6	A, B	16	A, B, C, D
7	A, C	17	A, C, D, E
8	A, D	18	A, B, D, E
9	A, E	19	A, B, C, E
10	A, B, C	20	A, B, C, D, E

A, B, C, D, and E represent topographic features, optical index features, texture features, SAR features, and original spectral features, respectively.

### 3.4. Classification algorithm and model construction

Random Forest (RF) is a classification and regression algorithm based on ensemble learning, proposed by Leo Breiman in 2001 [20]. It constructs multiple decision trees and aggregates their predictions through voting (for classification tasks) or averaging (for regression tasks), thereby enhancing the model’s generalization ability and robustness. RF has been widely applied in remote sensing classification due to its advantages in handling high-dimensional, nonlinear, and heterogeneous datasets.

In this study, RF was selected as the primary classifier because the objective was not only to obtain accurate land cover classification results but also to analyze the contribution of different remote sensing features. Compared with some other machine learning approaches, RF provides an effective feature importance evaluation mechanism based on out-of-bag (OOB) permutation importance, which facilitates the interpretation of model decision processes.

In this study, different feature sets were used to train the Random Forest (RF) model. First, the training dataset, feature variables, and class labels were input, and the model parameters were configured. The number of decision trees was determined through a sensitivity analysis combined with an out-of-bag (OOB) error convergence evaluation. Different numbers of decision trees (50, 100, 150, 200, 250, 300, 350, and 400) were tested, and the corresponding OOB errors were recorded. The results showed that the OOB error decreased rapidly with increasing tree numbers during the initial stage and gradually stabilized when the number of trees reached approximately 300 (S1 File). Meanwhile, the classification accuracy also showed a marginal improvement beyond 300 trees. Therefore, 300 decision trees were selected as the optimal parameter, providing a balance between classification performance and computational efficiency.

The bootstrap sampling strategy followed the classical bagging mechanism of Random Forest. During bootstrap sampling with replacement, approximately 63.2% of the original training samples are expected to be included in each decision tree, while the remaining samples are retained as out-of-bag (OOB) samples for internal model evaluation. The number of features randomly selected for node splitting (m_try) was determined according to the default strategy of the MATLAB TreeBagger implementation for classification tasks, where m_try was set to √p, with p representing the total number of input features in each feature combination scheme. Therefore, the actual m_try value varied among different feature combination experiments depending on their corresponding feature dimensions. Next, all features were integrated to perform an initial training of the RF classifier, yielding preliminary land cover classification results and feature importance scores. Based on these results, features with relatively low importance were removed, and different feature combinations were constructed for comparative experiments. The model was then retrained for refined classification, with particular emphasis on further distinguishing grassland types. Finally, feature importance and classification accuracy were jointly analyzed to evaluate the contribution of different features in the classification decision process. Based on this analysis, feature combinations were further optimized to enhance the performance of the classifier.

### 3.5. Accuracy assessment methods

Accuracy assessment was conducted based on the confusion matrix, from which overall accuracy (OA), producer’s accuracy (PA), user’s accuracy (UA), and the Kappa coefficient were calculated. The classification results obtained from different feature sets were compared to evaluate the effects of spectral features, SAR features, and their fusion on classification performance (Table 3).

**Table 3:** Description of accuracy metrics.

Metric	Formula	Note
Overall Accuracy (OA)	$OA=\frac{\sum_{i=\text{1}}^{n}x_{ii}}{N}$	i: class index; N total number of samples; x_ii_: number of correctly classified samples; n: number of classes
Producer’s Accuracy (PA)	${PA}_{i}=\frac{x_{ii}}{r_{i}}$	r_i_ total number of reference samples of class i
User’s Accuracy (UA)	${UA}_{i}=\frac{x_{ii}}{c_{i}}$	c_i_: total number of predicted samples of class i
Kappa Coefficient	$k=\frac{P_{o}-P_{e}}{\text{1}-P_{e}}$,where,P_o_ = OA,$P_{e}=\frac{\sum_{i=\text{1}}^{n}\left(r_{i}\times c_{i}\right)}{N^{\text{2}}}$	Po: observed agreement; P_e_: expected agreement by chance

### 3.6. Time-series analysis

To further reveal the temporal variation characteristics of grassland quantity and quality in the study area, the Random Forest model was trained using the constructed reference sample dataset described in Section 2.2.2. The trained model was subsequently applied to classify remote sensing data from 2015 to 2025, enabling long-term grassland information extraction. After extracting grassland-specific information for each year, fractional vegetation cover (FVC) was introduced as a key indicator for evaluating grassland quality, and a slope-based trend analysis was conducted on FVC. FVC represents the proportion of ground area covered by the vertical projection of vegetation and is closely related to vegetation growth status, canopy density, and ecological quality. Therefore, it plays an important role in grassland ecological monitoring. In this study, the Raster Calculator in ArcGIS was used to analyze the spatiotemporal variation of FVC from 2015 to 2025 and to determine its dominant temporal trends, thereby estimating the average rate of increase or decrease in vegetation cover over time.

In this study, fractional vegetation cover (FVC) was estimated based on the Pixel Dichotomy Model (PDM), which assumes that each pixel can be represented as a linear combination of a pure vegetation endmember and a pure soil endmember [21]. The fractional vegetation cover (FVC) is calculated as follows:


$$\begin{array}{c} FVC=\frac{NDVI-{NDVI}_{soil}}{{NDVI}_{veg}-{NDVI}_{soil}} \end{array}$$ (1)

where *NDVI*_*soil*_ and *NDVI*_*veg*_ represent the NDVI values of pure soil and pure vegetation endmembers, respectively. To ensure the robustness of the endmember selection, the percentile-based method was adopted in this study. Considering that NDVI distributions may vary among different years due to vegetation phenology, climatic conditions, and differences among Landsat sensors, the soil and vegetation endmembers were determined separately for each available observation year rather than using fixed values throughout the entire study period. Specifically, the 5th percentile of annual NDVI distribution within the study area was used as the soil endmember, while the 95th percentile was used as the vegetation endmember, expressed as follows:


$$\begin{array}{c} {NDVI}_{soil,y}={NDVI}_{y(\text{5}\%)},{NDVI}_{veg,y}={NDVI}_{y(\text{9}\text{5}\%)} \end{array}$$ (2)

where *NDVI*_*soil,y*_ and *NDVI*_*veg,y*_ represent the soil and vegetation endmembers derived from the NDVI distribution of year y, respectively.

To ensure the physical validity of the results, FVC values were constrained within the range of 0–1:


$$\begin{array}{c} FVC=\text{min}(\text{1},\text{max}(\text{0},FVC)) \end{array}$$ (3)

To evaluate the reliability of the PDM-derived FVC estimates, field-measured FVC data collected during 2021–2024 were compared with the corresponding estimated FVC values at the survey locations. The comparison showed that the estimated FVC values generally agreed with field observations, indicating the applicability of the Pixel Dichotomy Model for characterizing vegetation coverage in the study area. However, due to the limited number of field observations, this validation was mainly used as a qualitative assessment rather than a comprehensive statistical evaluation.

Equation (4) presents the calculation formula for the slope-based trend analysis [22]:


$$\begin{array}{c} Slope=\frac{n\sum_{i=\text{1}}^{n}\left(i\times FVC_{i}\right)-\sum_{i=\text{1}}^{n}i\times \sum_{i=\text{1}}^{n}FVC_{i}}{n\sum_{i=\text{1}}^{n}i^{\text{2}}-{\left(\sum_{i=\text{1}}^{n}i\right)}^{\text{2}}} \end{array}$$ (4)

where *Slope* represents the slope of the regression equation for each pixel, *FVC*_*i*_ denotes the fractional vegetation cover in the *i*-th year, and *n* is the total number of years in the study period. When *Slope* > 0, the FVC of the pixel exhibits an increasing trend; when *Slope* = 0, the FVC remains relatively stable; and when *Slope* < 0, the FVC shows a decreasing trend.

Based on the aforementioned methods, multi-temporal FVC observations for selected cloud-free years between 2000 and 2025 were obtained. Subsequently, a joint analysis was conducted by integrating grassland extraction results with annual FVC data. Changes in grassland area reflect the expansion and contraction of grassland quantity, while the temporal variation of FVC further characterizes vegetation growth status, canopy density, and the succession dynamics of grassland quality. On this basis, the slope of FVC change was introduced to quantitatively assess the long-term trend of vegetation cover. The direction and magnitude of the slope were used to determine the degree of improvement or degradation in grassland conditions, thereby identifying the direction and intensity of ecological changes across different regions. By integrating grassland area, FVC levels, and their temporal trends, this study establishes a multidimensional monitoring framework for grassland dynamics in the study area from 2000 to 2025, providing a more systematic and reliable scientific basis for grassland ecological conservation, degradation restoration, and resource management.

### 3.7. Correlation analysis

To quantitatively analyze the correlation and variation characteristics between fractional vegetation cover (FVC) and its influencing factors, both the Pearson correlation coefficient and the Spearman rank correlation coefficient were employed to assess the relationships between variable pairs. Among them, the Pearson correlation coefficient is used to characterize the linear relationship between variables, and its calculation formula is given as follows:


$$\begin{array}{c} r_{xy}=\frac{\sum_{i=\text{1}}^{n}\left(x_{i}-\overset{―}{x})(y_{i}-\overset{―}{y}\right)}{\sqrt{\sum_{i=\text{1}}^{n}{\left(x_{i}-\overset{―}{x}\right)}^{\text{2}}\sum_{i=\text{1}}^{n}{\left(y_{i}-\overset{―}{y}\right)}^{\text{2}}}} \end{array}$$ (5)

where *x*_*i*_ and *y*_*i*_ represent the values of the two variables at the *i*-th sample, respectively; $\overset{―}{x}$ and $\overset{―}{y}$ denote the mean values of the variables; *n* is the number of samples; and *r*_*xy*_ is the Pearson correlation coefficient. The value of *r*_*xy*_ ranges from −1–1, with values closer to 1 in absolute terms indicating a stronger correlation.

To further characterize the monotonic relationship between variables and reduce the influence of outliers, the Spearman rank correlation coefficient was introduced in this study, and its calculation formula is given as follows:


$$\begin{array}{c} \rho =\text{1}-\frac{\text{6}\sum_{i=\text{1}}^{n}d_{i}^{\text{2}}}{n(n^{\text{2}}-\text{1})} \end{array}$$ (6)

where *d*_*i*_ represents the difference between the ranks of the two variables for the *i*-th sample, and *ρ* denotes the Spearman correlation coefficient, which also ranges from −1–1. By combining Pearson and Spearman correlation analyses, the relationships between grassland FVC and its potential influencing factors can be comprehensively evaluated from both linear and monotonic perspectives. These statistical analyses were primarily used to identify potential associations among variables rather than to establish direct causal mechanisms. Therefore, the results should be interpreted as statistical evidence of relationships between FVC and environmental factors, while the underlying mechanisms require further investigation using multivariate statistical models or interpretable machine learning approaches.

## 4. Results and discussion

### 4.1. Grassland extraction results and accuracy assessment

The land cover classification results based on different feature combination schemes are shown in Fig 3, including six classes: built-up land, cropland, water bodies, forest, bare land, and grassland. Table 4 presents the number of grassland pixels extracted under different feature combinations, while Table 5 summarizes the classification accuracy of land cover and the user’s accuracy (UA) and producer’s accuracy (PA) for the grassland class under each feature combination scheme.

**Table 4:** Grassland extraction results under different feature combination schemes.

Scheme	Feature Combination	Number of Grassland Pixels	Area (km²)	Scheme	Feature Combination	Number of Grassland Pixels	Area (km²)
Ground Truth	–	64069	51.82	Ground Truth	–	64069	51.82
1	A	61643	49.86	11	ABD	63513	51.37
2	B	61890	50.06	12	ABE	62576	50.61
3	C	15011	12.14	13	ACD	57632	46.61
4	D	47407	38.34	14	ACE	61749	49.94
5	E	0	0	15	ADE	56539	45.73
6	AB	62657	50.68	16	ABCD	63331	51.22
7	AC	60896	49.25	17	ACDE	57476	46.49
8	AD	56577	45.76	18	ABDE	62708	50.72
9	AE	60613	49.03	19	ABCE	62911	50.88
10	ABC	62932	50.90	20	ABCDE	63431	51.31

A, B, C, D, and E represent topographic features, optical index features, texture features, SAR features, and original spectral features, respectively.

**Table 5:** Comparison of land cover classification accuracy under different feature combination schemes.

Scheme	Feature Combination	OA/%	Kappa Coefficient	UA/%	PA/%	F1	Scheme	Feature Combination	OA/%	Kappa Coefficient	UA/%	PA/%	F1
1	A	87.27	0.2533	56.76	58.76	57.74	11	ABD	93.58	0.7059	93.70	94.91	94.30
2	B	92.74	0.6584	89.51	93.20	91.32	12	ABE	93.44	0.6979	92.67	95.30	93.97
3	C	86.63	0.0855	21.13	97.46	34.72	13	ACD	90.96	0.0253	85.58	96.03	90.51
4	D	88.74	0.3224	69.87	96.89	81.13	14	ACE	89.36	0.4043	80.57	83.16	81.84
5	E	85.96	0	0	0	0	15	ADE	90.73	0.5079	83.64	95.59	89.23
6	AB	93.43	0.6976	92.49	94.98	93.72	16	ABCD	93.76	0.7154	93.76	95.19	94.47
7	AC	88.13	0.4028	80.37	84.29	82.28	17	ACDE	90.97	0.5361	85.34	96.01	90.36
8	AD	90.69	0.5053	83.65	95.70	89.27	18	ABDE	93.53	0.7032	92.76	95.18	93.95
9	AE	87.28	0.2526	56.19	59.21	57.65	19	ABCE	93.65	0.7093	93.32	95.37	94.34
10	ABC	93.67	0.7105	93.16	95.17	94.15	20	ABCDE	93.79	0.7165	94.00	95.23	94.61

**Fig 3:**
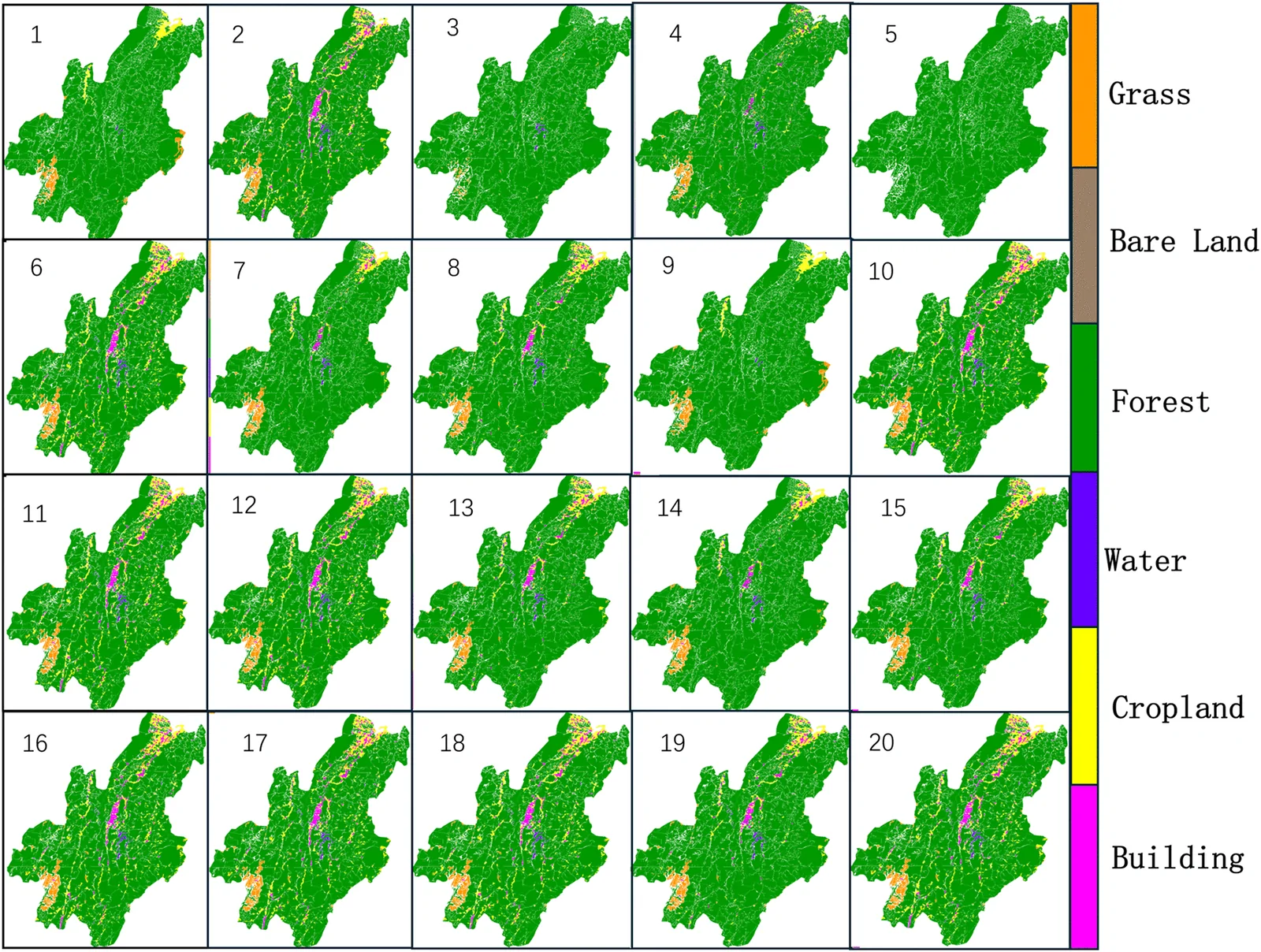
Land cover types of chengbu county in 2025 under different feature combination schemes.

From the perspective of grassland extraction results (Table 4), significant differences were observed among different feature combinations, indicating that the selection of input features directly affects the ability of the classifier to identify grassland. Among the single-feature schemes (Schemes 1–5), A, B, C, and D were capable of extracting different amounts of grassland information, whereas Scheme 5 (E, original spectral features) failed to identify grassland pixels, resulting in zero extracted area. This indicates that original spectral information alone is insufficient for grassland discrimination in the study area. The possible reason is that, in subtropical mountainous regions, grasslands are often distributed as fragmented patches with heterogeneous vegetation density and complex background conditions. Consequently, grassland may exhibit spectral similarity with cropland, forest edges, and other low-cover vegetation types, while topographic effects and mixed pixels further increase spectral uncertainty. Therefore, relying only on original spectral bands may limit the capability of the classifier to capture grassland-specific characteristics.

Among the single-feature schemes, Scheme 2 (B, optical index features) extracted the largest grassland area (61,890 pixels, 50.06 km²), followed by Scheme 1 (A, topographic features) with 61,643 pixels (49.86 km²). Optical indices enhance vegetation-related information by integrating spectral responses from multiple bands, while SAR features provide additional structural and moisture-related information that is less affected by cloud contamination. Therefore, both feature types provide effective representations of grassland characteristics from different perspectives.

Compared with single-feature schemes, multi-feature combinations generally produced more consistent grassland extraction results. Scheme 11 (ABD) achieved the largest number of extracted grassland pixels (63,513 pixels, 51.37 km²), while Scheme 20 (ABCDE) extracted 63,431 pixels (51.31 km²). Although Scheme 20 incorporated all available feature types, its extracted grassland area was slightly lower than Scheme 11. This suggests that additional features may not necessarily increase the detected grassland extent but can improve classification stability by reducing confusion among different land cover categories.

Regarding classification accuracy (Table 5), different feature combinations exhibited clear differences. Among the single-feature schemes, Scheme 2 (B, optical index features) achieved the best performance, with an OA of 92.74% and a Kappa coefficient of 0.6584. This demonstrates the important role of vegetation indices in grassland identification because they are closely related to vegetation growth status, canopy conditions, and biomass variation. In contrast, Scheme 5 (E, original spectral features) obtained a Kappa coefficient of 0 and UA and PA values of 0%, indicating that original spectral bands alone cannot effectively distinguish grassland from other land cover types in the study area.

The integration of multiple feature types further improved classification performance. Among the dual-feature schemes, Scheme 6 (AB) achieved the highest accuracy, with an OA of 93.43% and a Kappa coefficient of 0.6976. Compared with optical indices alone (Scheme 2), the combination of SAR and optical information increased the Kappa coefficient by 0.0392. This improvement indicates that SAR features provided complementary information beyond optical characteristics. Because SAR signals are sensitive to vegetation canopy structure, surface roughness, and moisture conditions, they can provide additional discrimination information for grassland areas with similar spectral responses, thereby reducing confusion among vegetation-covered land types. However, the improvement introduced by SAR features was relatively limited compared with optical-texture combinations, possibly because the fragmented distribution and strong spatial heterogeneity of mountainous grasslands cannot be fully characterized by backscatter information alone.

For three-feature combinations, Scheme 10 (ABC) and Scheme 11 (ABD) achieved OA values of 93.67% and 93.58%, with Kappa coefficients of 0.7105 and 0.7059, respectively. The results demonstrate that combining different feature groups can effectively improve grassland separability. In mountainous regions, terrain conditions strongly influence vegetation distribution and spectral characteristics. Therefore, terrain-related variables can compensate for the limitations of spectral information and improve classification performance.

Among the four-feature combinations, Scheme 16 (ABCD) achieved an OA of 93.76% and a Kappa coefficient of 0.7154. Among all evaluated feature combinations, Scheme 20 (ABCDE) achieved the highest OA (93.79%) and Kappa coefficient (0.7165). However, the difference compared with Scheme 16 (OA = 93.76%, Kappa = 0.7154) was relatively small, indicating that the additional features provided limited incremental improvement under the current experimental conditions. Nevertheless, the results suggest that integrating complementary information from spectral, structural, spatial, and environmental dimensions can improve classification robustness. Among these feature categories, texture information played an important role in improving grassland discrimination because it describes spatial heterogeneity and landscape organization that cannot be captured by pixel-based spectral features alone. Mountainous grasslands in the study area are typically characterized by fragmented patches, uneven vegetation coverage, and transitional boundaries with forests and croplands. Texture features can capture these spatial differences by representing local variations in vegetation distribution and canopy patterns, which explains their additional contribution to classification performance. Similar advantages of multi-source remote sensing fusion have also been reported in recent grassland monitoring studies, where integrating heterogeneous remote sensing information improved the representation of vegetation characteristics and enhanced model performance.

The classification accuracy obtained in this study is comparable to previous remote sensing-based grassland and land-cover mapping studies conducted in mountainous and heterogeneous environments. Previous studies have reported that grassland classification in mountainous regions is generally challenging due to spectral confusion among grassland, forest edges, cropland, and other vegetation types, with overall accuracies commonly ranging from approximately 85% to 95% depending on data sources [23], classification algorithms, and landscape complexity. The OA of 93.79% achieved in this study is therefore within the high-performance range reported by previous studies, indicating that the integration of optical, SAR, texture, and topographic features effectively improves grassland discrimination under subtropical mountainous conditions.

The grassland-specific accuracy assessment further confirmed the effectiveness of feature fusion. To provide a more comprehensive evaluation of grassland classification performance, the F1-score was calculated based on UA and PA, providing a balanced evaluation by integrating both commission and omission errors. Among the single-feature schemes, Scheme 2 achieved relatively high grassland UA and PA values (89.51% and 93.20%, respectively), with an F1-score of 91.32%, demonstrating its ability to achieve a relatively balanced classification performance for grassland identification. After integrating additional features, the classification reliability was further improved. Scheme 6 increased UA and PA to 92.49% and 94.98%, respectively, resulting in an F1-score of 93.72%. Among all feature combinations, Scheme 20 achieved the highest grassland UA (94.00%), PA (95.23%), and F1-score (94.61%), indicating the most balanced performance among the evaluated feature combinations. However, the difference between Scheme 20 and Scheme 16 (F1-score of 94.47%) was relatively small, suggesting that the contribution of additional features became limited when sufficient complementary information had already been incorporated. Although the overall accuracy exceeded 93%, the Kappa coefficients remained around 0.71. This discrepancy may be related to the imbalanced distribution of land-cover categories and the dominance of forest areas in the mountainous study region, where major classes contribute more substantially to overall accuracy. In addition, spectral and structural similarities among grassland, cropland, and forest edge areas may introduce classification uncertainty. Therefore, OA, Kappa, UA, PA, and F1-score should be jointly considered for a comprehensive assessment of grassland extraction performance. These results indicate that multi-source feature fusion not only improves overall classification accuracy but also enhances the completeness and reliability of grassland identification.

Overall, the variations in grassland UA, PA, and F1-score were consistent with the trends of OA and Kappa coefficient, indicating that the improvements obtained from multi-feature fusion were reflected in both overall classification performance and grassland-specific identification capability. The results indicate that different feature types contribute differently to grassland extraction: optical indices mainly characterize vegetation physiological conditions, SAR features provide complementary information on canopy structure and surface conditions, texture features describe spatial heterogeneity and patch characteristics, and terrain-related variables reflect environmental constraints on vegetation distribution. The improved performance of multi-feature combinations demonstrates that grassland identification in subtropical mountainous regions requires the joint representation of spectral, structural, spatial, and environmental characteristics rather than reliance on a single information source.

### 4.2. Feature importance analysis

The feature importance analysis based on the out-of-bag (OOB) permutation importance method was conducted to quantify the contribution of different variables to the Random Forest classification process and to improve the interpretability of model decisions [24] As shown in Fig 4, considerable differences were observed among input features, indicating that different remote sensing variables provided complementary information for grassland discrimination

**Fig 4:**
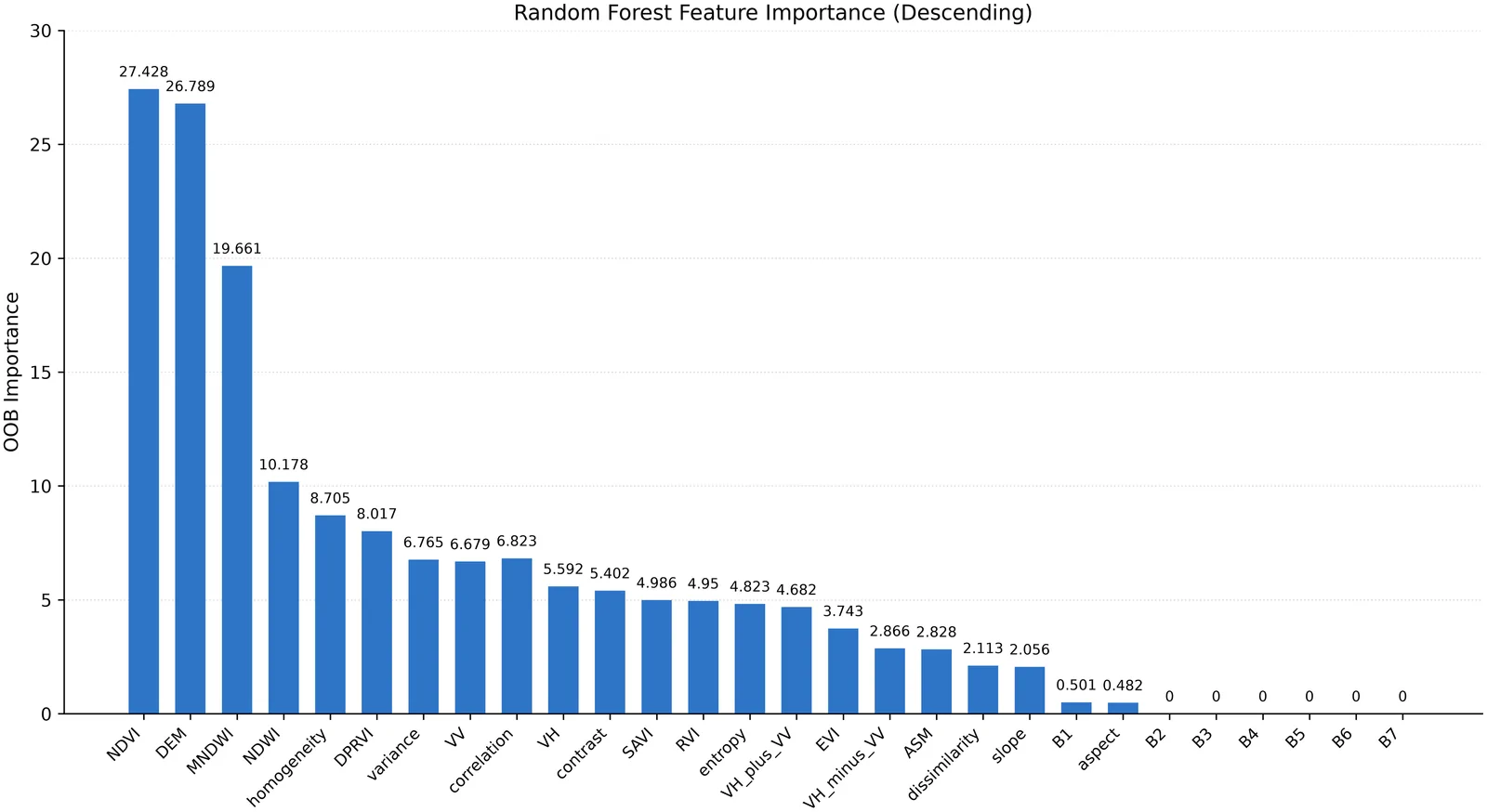
Feature importance.

Among all features, NDVI exhibited the highest importance value (27.428), followed by DEM (26.789), MNDWI (19.661), NDWI (10.178), homogeneity (8.705), and DpRVI (8.017). The dominance of vegetation indices and terrain variables indicates that grassland identification in the subtropical mountainous region was primarily controlled by vegetation conditions and environmental gradients. Similar findings have been reported in mountainous grassland and ecological remote sensing studies, where vegetation indices and topographic variables were identified as key predictors because they effectively characterize vegetation status and habitat constraints.

NDVI showed the greatest contribution among all variables, highlighting the importance of vegetation physiological characteristics in grassland classification. NDVI integrates information related to chlorophyll activity, vegetation density, and canopy development, which enables effective discrimination between grassland and other vegetation-covered land types. Similar conclusions have been reported in previous grassland mapping studies, where NDVI and related vegetation indices were consistently ranked among the most important features due to their direct relationship with vegetation coverage and productivity.

DEM ranked second in this study, which differs from some low-relief region studies but agrees with findings from mountainous ecosystems. In complex terrain environments, elevation gradients regulate temperature, precipitation distribution, soil moisture, and vegetation composition, resulting in strong ecological differentiation among land-cover types. Therefore, the high contribution of DEM indicates that topographic information provides important environmental constraints for grassland distribution in mountainous landscapes.

Moisture-related indices, including MNDWI and NDWI, also showed relatively high importance. Although originally designed for water-body detection, these indices can characterize surface moisture conditions and vegetation water availability. Their high ranking suggests that moisture variability contributes to distinguishing grasslands from croplands, forest edges, and other vegetation types with similar spectral characteristics. Similar applications of moisture-related indices have also demonstrated their value in vegetation monitoring and ecosystem classification under humid environments.

Texture features showed moderate importance, particularly homogeneity, demonstrating the value of spatial information in fragmented mountainous landscapes. Previous studies have indicated that texture features can improve land-cover classification by capturing spatial heterogeneity and patch structure, especially where grasslands are distributed as fragmented patches with strong boundary effects [25].

SAR-related variables, including VH, VV, and vegetation radar indices, showed lower importance compared with optical indices and terrain variables. However, SAR features still contributed complementary structural and moisture information, improving classification robustness under cloudy and humid conditions. Similar results have been observed in multi-source remote sensing studies, where SAR data generally provided auxiliary information rather than dominant predictors for vegetation classification.

In contrast, original spectral bands exhibited relatively limited contributions, suggesting that directly using raw reflectance information may not sufficiently capture the ecological characteristics of mountainous grasslands. Derived variables, including vegetation indices, moisture indices, and texture features, enhanced feature representation by integrating physiological, structural, and environmental information.

Overall, the feature importance ranking in this study followed the general pattern of vegetation indices and terrain variables > moisture and texture features > SAR features > original spectral bands. This pattern is generally consistent with previous grassland and mountainous ecosystem studies, confirming that vegetation conditions and topographic constraints are the dominant factors controlling grassland discrimination. Meanwhile, the integration of multi-source remote sensing features improves classification performance by incorporating complementary ecological information from spectral, structural, spatial, and environmental perspectives [26].

### 4.3. Temporal analysis of grassland changes

To investigate the long-term dynamics of grassland resources, the Random Forest classification model trained using the constructed reference sample dataset was applied to generate multi-temporal land-cover maps for selected years between 2000 and 2025. For the period of 2015–2025, classification was performed using the five-feature combination dataset, whereas the 2000–2011 classification was based on the four-feature combination dataset due to the absence of SAR observations. It should be noted that the classifications for 2000–2011 were obtained through temporal transfer of the model trained with recent reference samples. Due to the lack of corresponding historical field observations, independent validation of these reconstructed maps was not conducted. Therefore, the results for this period should be regarded as indicative representations of historical land-cover patterns rather than fully validated classification products. The spatial distribution of land-cover types during the study period is presented in Fig 5. Based on the classification results, grassland area and fractional vegetation cover (FVC) were calculated to characterize changes in grassland quantity and ecological quality, respectively (Fig 6 and Fig 7).

**Fig 5:**
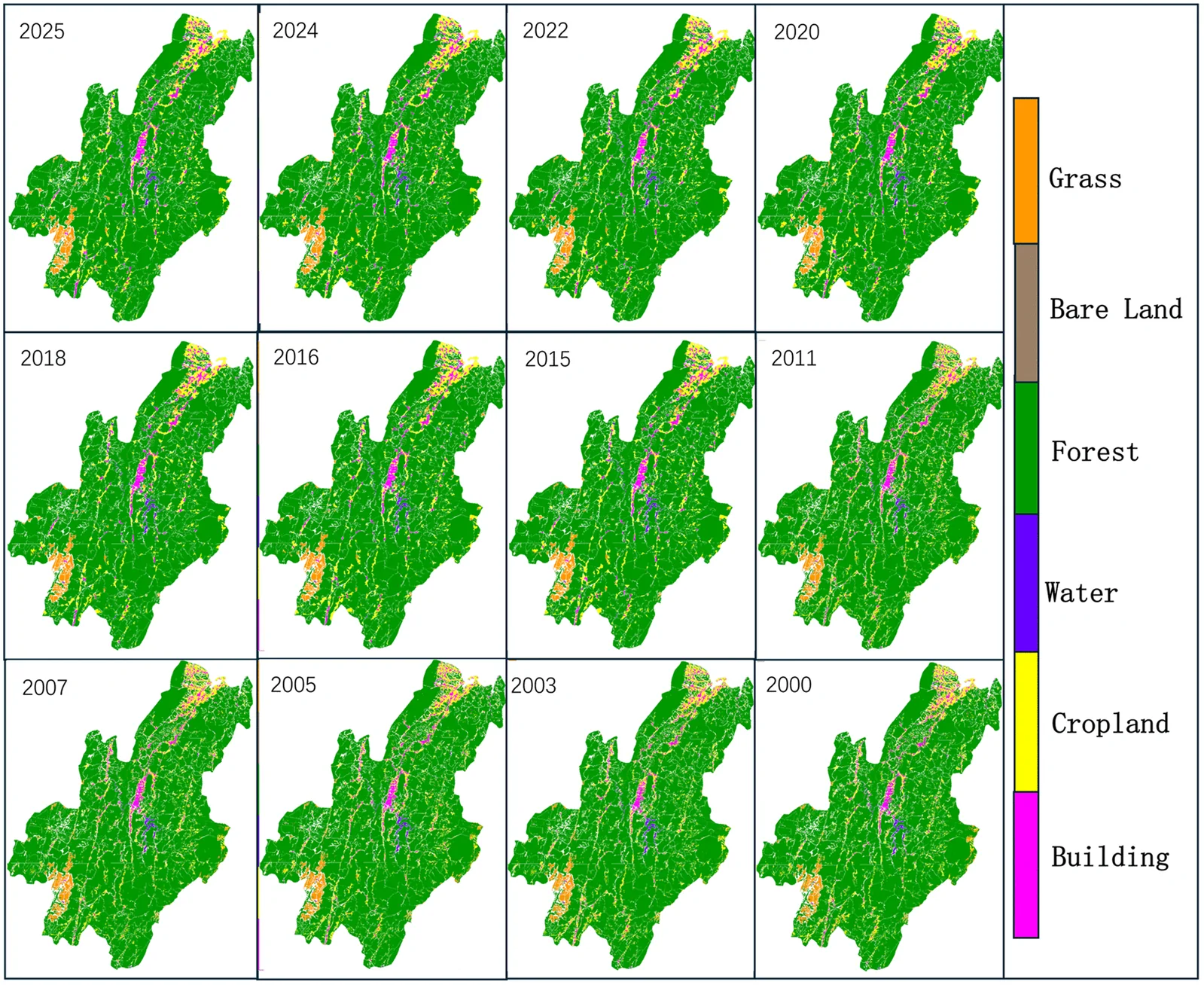
Land cover types for each year.

**Fig 6:**
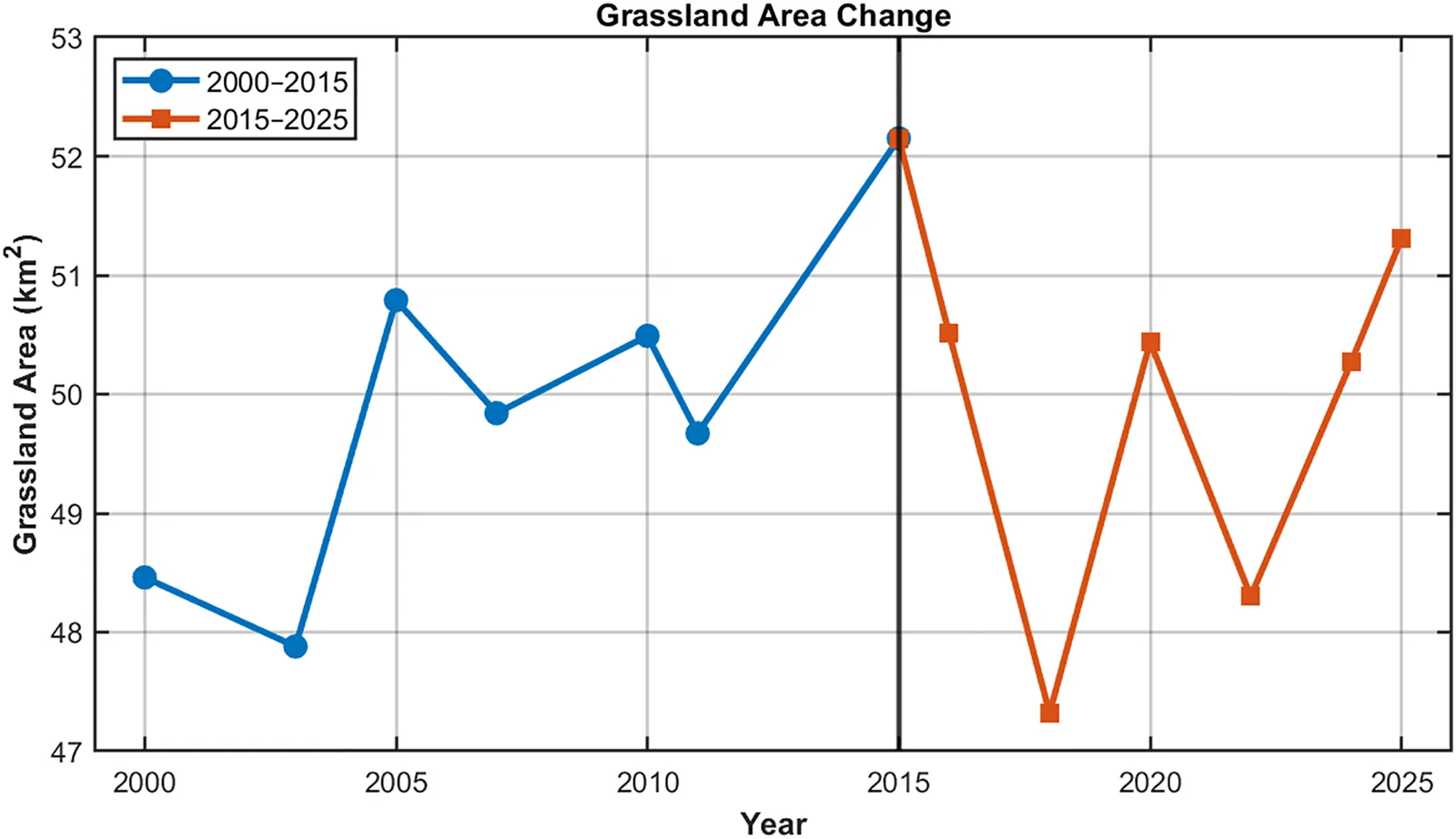
Changes in grassland area.

**Fig 7:**
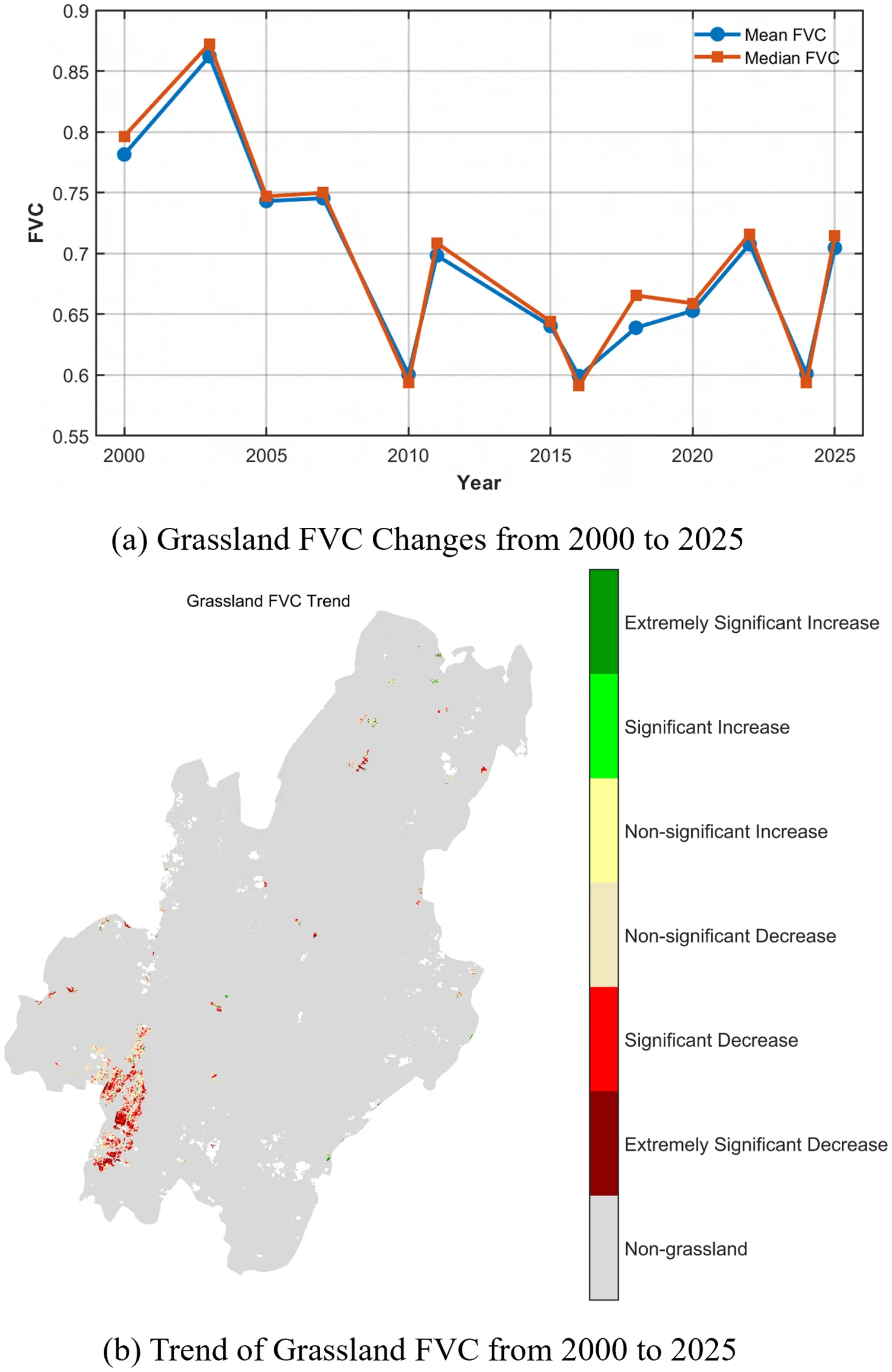
Grassland Fractional Vegetation Cover (FVC) and its change trends for each year.

The results indicate that grassland area remained relatively stable during 2000–2025, although interannual fluctuations occurred. During 2000–2011, grassland area ranged from 47.88 to 50.79 km², reaching the minimum value in 2003 and the maximum value in 2005, followed by a relatively stable period. During 2015–2025, grassland area exhibited greater fluctuations, decreasing from 52.15 km² in 2015 to 47.32 km² in 2018, and subsequently recovering to 50.44 km² in 2020 and 51.31 km² in 2025. Despite these variations, the overall change magnitude remained limited, indicating that grassland resources did not experience a persistent expansion or degradation trend. Furthermore, the Mann–Kendall (MK) test and Sen’s slope estimator were applied to evaluate the significance of long-term grassland area changes. The results showed that the trend was not statistically significant (p > 0.05), and the small Sen’s slope value further confirmed the overall stability of grassland extent. Therefore, the observed changes mainly represent short-term fluctuations rather than directional long-term changes.

The temporal variations in grassland area should be interpreted considering both ecological processes and remote sensing uncertainties. In mountainous ecosystems, variations in remotely sensed grassland extent may result not only from actual land-cover conversions but also from changes in vegetation phenology, canopy conditions, and spectral responses caused by environmental variability. Differences in precipitation, temperature, and vegetation growth stages among years may influence the spectral separability between grassland and adjacent land-cover types, particularly in transition zones involving forests, croplands, and sparse vegetation. In addition, local management activities, including grazing practices and ecological restoration measures, may influence vegetation composition and coverage conditions. Therefore, the observed grassland area fluctuations likely reflect the combined effects of environmental variability, land management, and ecosystem responses.

To further assess grassland ecological conditions, FVC was introduced as an indicator of vegetation coverage and growth status. The results showed that grassland FVC exhibited an overall fluctuating pattern during 2000–2025, with relatively stable interannual variations at the regional scale. The Mann–Kendall (MK) test and Sen’s slope estimator were applied to quantitatively evaluate the long-term FVC trend. The results showed that the trend of mean FVC was not statistically significant (MK Z = −0.82, p = 0.41), with a Sen’s slope of −0.0013 per year, indicating a slight decreasing trend but no significant long-term change. Although FVC decreased during 2000–2005 and subsequently recovered with fluctuations after 2005, these variations mainly represented interannual variability rather than a persistent directional trend. However, the stability of regional mean FVC does not indicate that all grassland areas maintained unchanged ecological conditions, as significant spatial heterogeneity existed at the local scale. Some areas, particularly the southwestern part of Nanshan Pasture, exhibited significant decreasing trends in FVC, suggesting localized vegetation degradation despite overall regional stability.

The spatial distribution of FVC trends revealed heterogeneous ecological responses within the study area. Most grassland regions showed non-significant trends, suggesting that vegetation conditions were generally maintained at the regional scale. However, significant increasing and decreasing trends were observed in some localized areas, indicating substantial spatial heterogeneity in grassland ecological responses. In particular, the southwestern part of Nanshan Pasture exhibited significant decreasing trends in FVC, suggesting potential vegetation degradation risks in this high-quality grassland region. Regions with increasing FVC may be associated with vegetation recovery, improved pasture management, and reduced disturbance intensity. Conversely, areas with declining FVC were mainly distributed in regions experiencing stronger human disturbances. Field investigations indicated that grazing activities and tourism development may have increased ecological pressure in certain areas. Continuous grazing can reduce vegetation regeneration capacity and alter plant community structures, whereas tourism activities may cause vegetation trampling and habitat fragmentation. However, these influences were mainly inferred from field observations and their spatial consistency with FVC changes, and their individual contributions cannot be quantitatively separated due to the lack of long-term human activity datasets.

The combined analysis of grassland area and FVC indicates that the Nanshan Pasture maintained relatively stable grassland extent during 2000–2025, while vegetation conditions exhibited spatially heterogeneous changes. Most grassland areas showed non-significant FVC trends, suggesting that vegetation conditions were generally maintained at the regional scale. However, significant decreasing trends were detected in localized areas, particularly in the southwestern part of the pasture, indicating potential ecological degradation risks in specific regions. These results suggest that stable grassland extent does not necessarily represent stable ecological conditions, as grasslands may maintain their spatial distribution while experiencing reductions in vegetation coverage and ecological quality. The localized FVC declines may reflect weakened vegetation growth conditions or increased ecological stress caused by environmental variability and human disturbances. Therefore, both grassland extent and vegetation quality indicators should be jointly considered for comprehensive grassland ecological assessment. Future studies should integrate quantitative climate data, grazing intensity records, tourism activity datasets, and high-resolution land-use information to further clarify the mechanisms driving grassland ecological changes in subtropical mountainous regions.

### 4.4. Correlation analysis

In this study, the Nanshan pasture region was selected as the primary study area. Based on the human activity intensity proxy dataset constructed in Section 2.2.4, the influence of anthropogenic activities on grassland FVC was further analyzed. The dataset integrates information on the spatial distribution and extent of residential areas, as well as the accessibility effects of major roads. Together with DEM, slope, and aspect factors, correlation analyses were conducted to investigate the relationship between anthropogenic activities, topographic conditions, and grassland FVC. The results are presented in Table 6 and Fig 8.

**Table 6:** Correlation analysis between topographic factors, human activities, and grassland FVC.

	Pearson		Spearman	
	Correlation (r)	p-value	Correlation (ρ)	p-value
DEM	−0.2839	< 0.001	−0.2700	< 0.001
Slope	0.0411	< 0.001	0.0087	< 0.001
Aspect(sin)	0.0370	< 0.001	0.0402	< 0.001
Aspect(cos)	0.0402	< 0.001	0.0277	< 0.001
Human Activity	−0.1563	< 0.001	−0.1678	< 0.001

**Fig 8:**
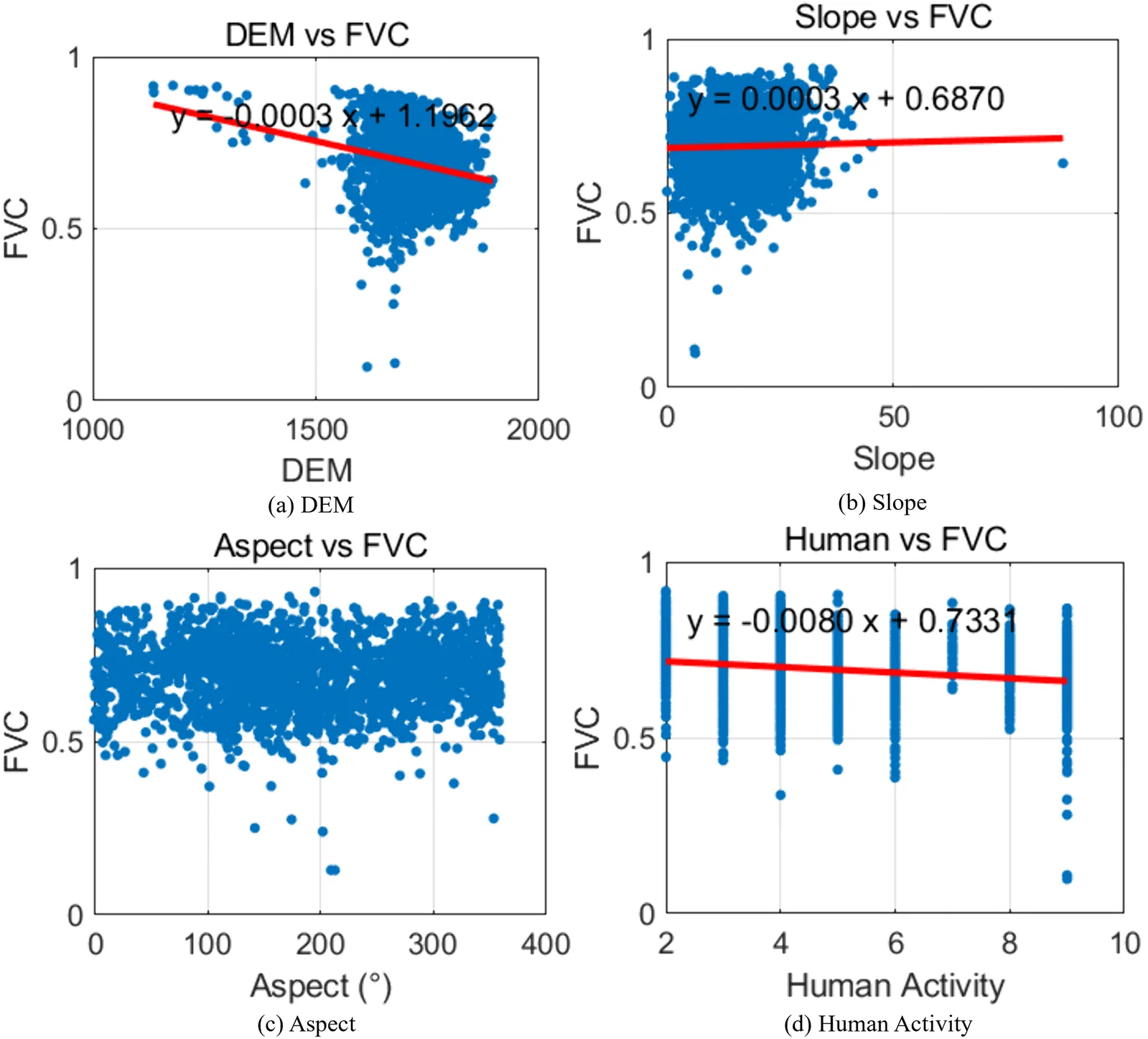
Correlation analysis between topographic factors, human activities, and grassland FVC.

The correlation analysis results indicate that grassland FVC is influenced by multiple environmental and anthropogenic factors, with different factors showing varying degrees of contribution (Table 6 and Fig 8). Among the analyzed variables, DEM exhibited the strongest relationship with FVC, showing significant negative correlations in both Pearson (r = −0.2839, p < 0.001) and Spearman analyses (r = −0.2700, p < 0.001). This indicates that elevation represents an important environmental gradient associated with grassland vegetation conditions. The negative relationship may be related to the combined effects of lower temperatures, shorter growing seasons, and changes in vegetation distribution patterns along elevation gradients in mountainous regions. Therefore, elevation-related environmental constraints may partly explain the spatial variability of grassland FVC.

Human activity intensity also showed a significant negative correlation with grassland FVC (Pearson r = −0.1563, Spearman r = −0.1678, p < 0.001), although the correlation strength was weaker than that of elevation. This result suggests that anthropogenic disturbance has a measurable but relatively limited impact on vegetation coverage at the regional scale. The negative relationship is consistent with field observations indicating increasing grazing and tourism activities in parts of the Nanshan pasture. Human disturbances, including grazing pressure and tourism-related vegetation trampling, may reduce vegetation regeneration capacity and contribute to localized FVC decline. However, the relatively weak correlation also indicates that the implementation of grassland conservation and management measures may have mitigated the overall impact of human activities.

In contrast, slope and aspect showed statistically significant but extremely weak relationships with FVC. The Pearson correlation coefficients of slope, aspect(sin), and aspect(cos) were 0.0411, 0.0370, and 0.0402, respectively, indicating limited explanatory power of micro-topographic factors for grassland vegetation coverage in the study area. Although these variables reached statistical significance due to the large number of sampled pixels, their low correlation coefficients suggest that slope and aspect played a relatively minor role compared with elevation and human activity intensity. This weak relationship may be related to the characteristics of the Nanshan pasture ecosystem, where a considerable proportion of grasslands are managed or artificial grasslands. Long-term pasture management practices, including vegetation restoration and grazing regulation, may have modified the natural relationship between vegetation distribution and local micro-topographic conditions. Consequently, slope and aspect exerted relatively limited influence on the spatial pattern of grassland FVC in the study area.

Climatic conditions are important driving factors influencing variations in grassland fractional vegetation cover (FVC). In this study, CHIRPS daily precipitation data and MODIS land surface temperature (LST) products were employed, with LST used as a proxy for air temperature. These datasets were selected because their temporal coverage satisfies the requirement for a continuous 25-year study period from 2000 to 2025, ensuring the completeness and consistency of the time series. Considering the relatively coarse spatial resolution of meteorological data (approximately 1–5 km), which is insufficient for pixel-scale correlation analysis, this study conducted the analysis at the regional scale. Specifically, monthly climate data during the growing season (March–September) were extracted for each year within the study area. LST was averaged to obtain monthly mean temperature, while precipitation data were first temporally accumulated and then used to calculate monthly mean precipitation. Subsequently, the mean temperature and average monthly precipitation during the growing season were derived. Based on these data, correlation analysis was performed between the climatic factors and the corresponding regional mean FVC of grasslands to evaluate the overall impact of climate variability on vegetation cover. The results are presented in Fig 9.

**Fig 9:**
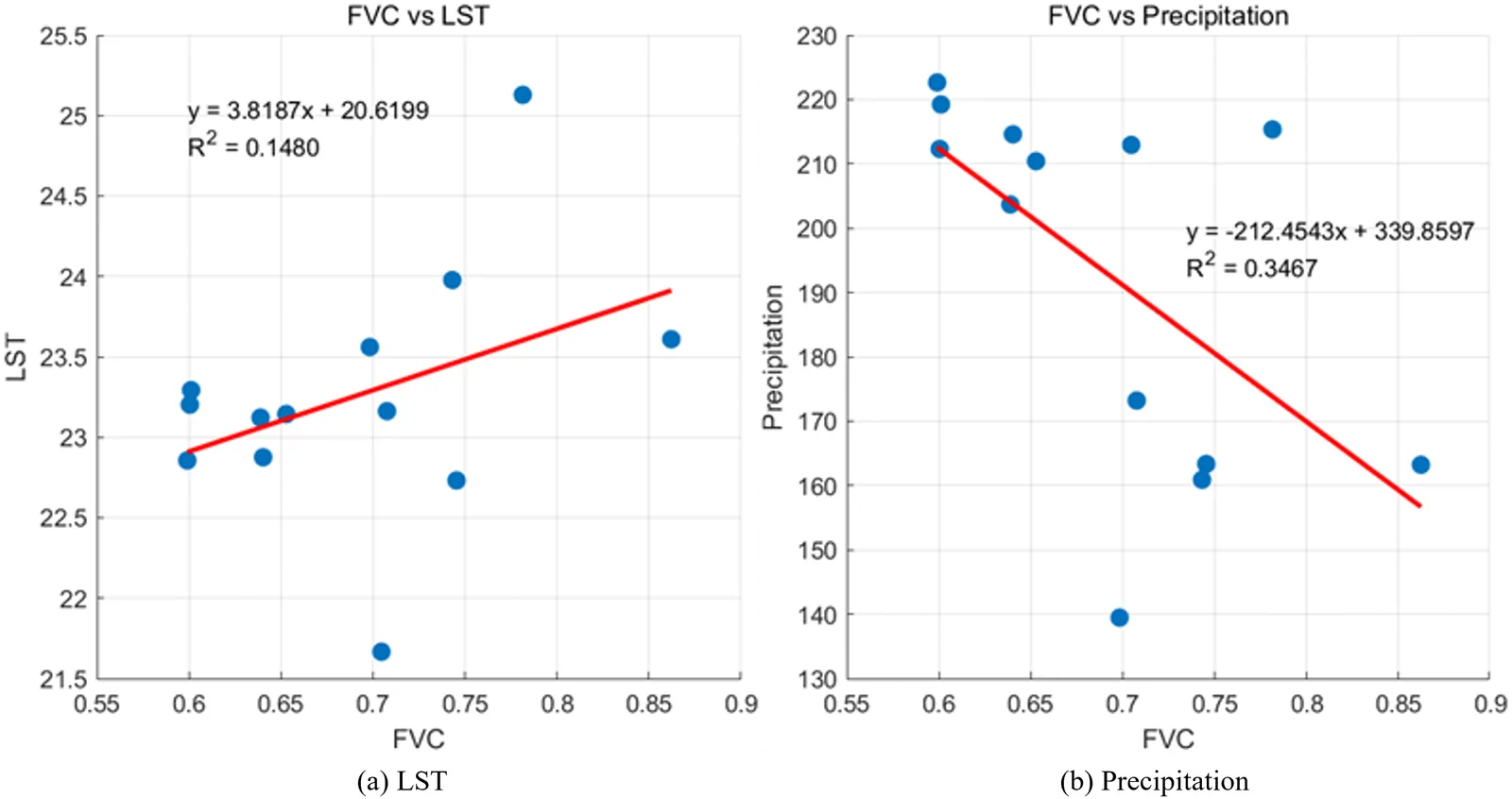
Correlation analysis between climatic factors and grassland FVC.

The correlation analysis indicates that grassland fractional vegetation cover (FVC) exhibits a weak positive correlation with land surface temperature (r = 0.385), but this relationship is not statistically significant (p > 0.05), with a low coefficient of determination (R² = 0.15). This suggests that LST has limited explanatory power for variations in grassland FVC. Considering the regional context, the Nanshan pasture is located in a humid subtropical climate zone, where thermal conditions are generally sufficient and show limited interannual variability. Therefore, interannual temperature variability was not a primary limiting factor controlling grassland FVC dynamics during the study period, which explains its insignificant influence on FVC dynamics.

In contrast, FVC shows a significant negative correlation with precipitation (r = −0.589, p < 0.05, R² = 0.35), indicating that precipitation variability is associated with interannual changes in grassland vegetation coverage. However, this negative relationship should not be interpreted as evidence that increased precipitation directly inhibits vegetation growth. In humid subtropical mountainous ecosystems, vegetation dynamics are generally controlled by multiple interacting environmental factors, including water availability, thermal conditions, solar radiation, soil properties, and human management activities. Therefore, the observed negative correlation may reflect the combined effects of climatic variability and ecosystem responses rather than a single limiting factor. In addition, the response of grassland vegetation to precipitation may vary depending on ecosystem conditions and management practices. In the Nanshan pasture, where managed and semi-natural grasslands coexist, grazing intensity, pasture management, and other anthropogenic disturbances may influence vegetation responses to climatic fluctuations. However, due to the lack of long-term observations of radiation conditions, soil moisture, and human activity intensity, the relative contributions of these factors cannot be quantitatively separated in this study. In addition, potential lag effects of precipitation and collinearity between precipitation and temperature/elevation were not quantitatively separated in this study. Therefore, the precipitation–FVC relationship identified here should be considered a statistical association rather than a direct causal mechanism, and further studies integrating multi-source ecological observations are required to better understand the driving processes of grassland vegetation dynamics.

Overall, precipitation showed a stronger statistical relationship with grassland FVC than temperature in the Nanshan pasture. However, grassland vegetation dynamics likely reflect the combined effects of climatic variability, environmental conditions, and anthropogenic disturbances, rather than the influence of a single climatic factor.

### 4.5. Limitations and future perspectives

Although this study achieved reliable grassland information extraction using multi-source remote sensing feature fusion, several limitations should be acknowledged.

(1) The reference samples were mainly generated through field survey data collected during 2021–2024 combined with visual interpretation of high-resolution imagery. Although this approach provides reliable training samples for characterizing the spectral and ecological properties of major land-cover categories, uncertainty may still exist due to interpretation subjectivity and temporal transferability issues. In this study, the visual interpretation was conducted by a single experienced interpreter following consistent interpretation criteria, and the potential uncertainty caused by subjective judgment cannot be completely eliminated. The application of these samples to historical Landsat imagery from 2000 onward assumes that the fundamental characteristics of relatively stable land-cover types, particularly mountainous grassland ecosystems, have remained comparable over time. This assumption is reasonable for the study area because Chengbu County is dominated by mountainous grassland landscapes with limited large-scale land-cover conversion. However, historical changes associated with ecological restoration programs, agricultural activities, and land management practices may still introduce uncertainties into retrospective classification results. Future studies could integrate multi-interpreter assessments, historical reference datasets, and multi-period field observations to further improve the reliability of long-term grassland monitoring. In addition, independent validation using historical reference data or archived high-resolution imagery is needed to further assess the accuracy of reconstructed land-cover maps for earlier periods.
(2) This study employed Random Forest as the primary classifier due to its robustness and interpretability for high-dimensional remote sensing features. However, emerging approaches, including gradient boosting algorithms and deep learning models, may provide additional opportunities for improving classification performance and capturing complex spatial patterns. Future research could conduct systematic comparisons among different machine learning and deep learning frameworks [27].
(3) Although multi-source feature fusion improved grassland classification accuracy, the contribution of individual features may vary across different environmental conditions. Future studies should further explore adaptive feature selection strategies and develop more transferable models for grassland monitoring across different geographic regions [28].
(4) Although correlation analyses were conducted to explore the relationships between grassland FVC and topographic, climatic, and anthropogenic factors, the complex interactions among multiple driving factors were not fully quantified in this study. The Pearson and Spearman correlation analyses mainly reveal statistical associations rather than causal relationships, and therefore cannot completely separate the individual contributions or interactions of different environmental variables. In addition, the influences of grazing intensity, tourism activities, and pasture management were inferred based on field observations and the spatial consistency between human activities and FVC variations, while long-term quantitative records of human disturbance were unavailable. Moreover, climate–vegetation relationships may be regulated by ecosystem management practices, particularly because managed and artificial grasslands account for a considerable proportion of grassland areas in the study region. Future studies should integrate long-term quantitative datasets, including grazing statistics, tourism intensity, soil moisture observations, and ecological monitoring data, and employ multivariate statistical approaches or interpretable machine learning methods (e.g., structural equation models and SHAP-based analysis) to further quantify the relative contributions and interactions among climate variability, environmental constraints, human disturbance, and grassland dynamics [29,30].
(5) Long-term grassland change analysis based on multi-temporal satellite observations may involve uncertainties related to sensor differences, FVC estimation, and error propagation. In this study, Landsat images from different sensors, including Landsat 5 TM, Landsat 8 OLI, and Landsat 9 OLI-2, were integrated to construct a long-term grassland monitoring dataset. Although consistent preprocessing procedures were applied to improve temporal comparability, differences in spectral response characteristics among sensors may still introduce uncertainties into long-term classification and FVC estimation. For FVC calculation, the 5th and 95th percentiles of NDVI were independently determined for each year to reduce the influence of interannual variations in vegetation conditions and environmental factors. In addition, field-measured FVC data collected during 2021–2024 were used for a preliminary evaluation of the PDM-derived FVC estimates, and the comparison indicated a general consistency between estimated and observed FVC values. However, due to the limited number of field observations, the validation was mainly qualitative, and uncertainties associated with NDVI endmember selection, vegetation phenology, and sensor transitions cannot be completely eliminated. These uncertainties may further propagate into long-term FVC trend analysis and ecological interpretation. Future studies should incorporate more extensive field measurements, quantitative validation approaches, cross-sensor calibrated datasets, and uncertainty assessment methods to further improve the reliability of long-term grassland monitoring.
(6) The accuracy assessment in this study was based on a random pixel-level division strategy, which may introduce potential spatial autocorrelation between training and validation samples due to the spatial continuity of remote sensing data. Neighboring pixels may share similar spectral, topographic, and textural characteristics, potentially leading to relatively optimistic accuracy estimates. Although the large number of reference pixels and consistent random sampling strategy improve the reliability of model evaluation, the influence of spatial dependence cannot be completely eliminated. Future studies should consider spatial cross-validation or spatially separated sampling strategies to provide more robust and conservative accuracy assessments.
(7) Although this study provided multi-temporal grassland classification maps, FVC products, and correlation analyses to characterize grassland dynamics, a comprehensive uncertainty quantification framework was not established for all outputs. The Random Forest model provides reliable classification results; however, pixel-level classification confidence maps were not generated in this study. Similarly, uncertainty intervals for correlation coefficients were not calculated. Therefore, uncertainties associated with model prediction, statistical estimation, and error propagation should be further considered when interpreting the results. Future studies should incorporate probabilistic classification methods, uncertainty mapping approaches, and statistical confidence interval analysis to establish a more comprehensive uncertainty assessment framework for long-term grassland monitoring [30].

## 5. Conclusions

This study systematically conducted grassland classification accuracy assessment, feature importance analysis, and grassland cover change monitoring from 2015 to 2025 based on multi-source remote sensing feature fusion and a Random Forest model. The main conclusions are as follows:

(1) Optical indices showed the best performance among single-feature schemes but remained insufficient for distinguishing grasslands from spectrally similar land-cover types. Multi-feature fusion significantly improved classification performance, with Scheme 11 achieving the largest grassland extraction area and Scheme 20 obtaining the highest accuracy (OA = 93.79%, Kappa = 0.7165). These results confirm the complementarity of spectral, optical index, SAR, texture, and topographic features, demonstrating the effectiveness of multi-source feature fusion for grassland mapping in complex mountainous environments.
(2) NDVI and DEM showed the highest contributions, indicating that vegetation conditions and topographic factors are the primary drivers of grassland classification. Vegetation and moisture indices (e.g., NDVI, MNDWI, and NDWI) provided essential spectral information, while texture features improved discrimination by capturing spatial heterogeneity. SAR features contributed complementary structural information and enhanced model robustness. Overall, multi-source feature integration improved grassland characterization, with spectral and topographic information playing dominant roles.
(3) The grassland area in Chengbu County remained relatively stable during 2000–2025, with no significant long-term expansion or contraction trend. However, stable grassland extent did not necessarily indicate completely stable ecological conditions. FVC exhibited temporal fluctuations and spatial heterogeneity during the study period, with most areas showing non-significant changes but localized decreasing trends observed in the southwestern part of Nanshan Pasture. These results suggest that grasslands may maintain their spatial distribution while experiencing localized variations in vegetation conditions. Therefore, long-term grassland monitoring should jointly consider changes in spatial extent and vegetation condition indicators to achieve a more comprehensive assessment of grassland dynamics.
(4) Correlation analysis revealed that grassland FVC dynamics in the Nanshan pasture were jointly associated with topographic, anthropogenic, and climatic factors. Elevation was identified as the most influential factor, indicating the important role of environmental gradients in regulating grassland vegetation patterns. Human activity showed a weaker negative association with FVC, while micro-topographic factors exhibited limited effects. Climatic factors, particularly precipitation, were significantly associated with FVC variation, but their influence should be interpreted cautiously due to the interactions among climate variability, ecosystem conditions, and management practices. Overall, grassland dynamics reflect the combined effects of natural constraints, human activities, and climatic variability.
(5) Long-term grassland monitoring based on multi-temporal satellite observations provides valuable insights into vegetation dynamics; however, uncertainties related to sensor differences, FVC estimation, and limited field validation should be considered. Although consistent preprocessing and FVC estimation strategies improved temporal comparability, further integration of cross-sensor calibration, extensive field observations, and uncertainty assessment methods is required to enhance the reliability of long-term grassland monitoring.

## Supporting information

S1 File Supplementary tables and figures. This file contains the confusion matrices for Scheme 2 and Scheme 20, as well as the sensitivity analysis of the number of decision trees.
(DOCX)
