# Supplementary material for: Multi-feature fusion for grassland information extraction and temporal change monitoring
Source: PLoS One. 2026 Nov 6;21(11):e0347821. doi: 10.1371/journal.pone.0347821 (PMC13742955; doi:10.1371/journal.pone.0347821)
Supplement: Supplementary file 1 [file pone.0347821.s002.pdf]

<u>Review of the Article submitted in PLOS One</u>

Article Title: Multi-Feature Fusion for Grassland Information Extraction and Temporal Change Monitoring

This study proposes a multi-feature fusion framework integrating optical, SAR, vegetation, texture, topographic, and time-series data within the Google Earth Engine platform, coupled with a Random Forest model, **to improve grassland mapping accuracy in complex mountainous terrain**. It further investigates the spatial and temporal dynamics of grasslands and identifies the relative influence of topographic, climatic, and anthropogenic factors on vegetation cover.

Article seems interesting. But it requires major revision for possible publication in the journal. The comments are as follows:

1. **The abstract does not clearly convey the motivation behind the study.** It should be revised to concisely highlight the research motivation, the novelty of the proposed methodology, its key findings, and the rationale for adopting the proposed approach.
2. **The authors state that "there are numerous challenges under complex surface conditions"; however, only the issue of short-term analysis has been discussed as a limitation of previous studies.** The other challenges should be clearly identified, explained, and supported with appropriate justification.
3. **The novelty of the proposed methodology is not clearly articulated.** The specific methodological contribution and its significance should be explicitly stated at the end of the Introduction.
4. **Section 2.2 states that grassland survey data obtained from field measurements were used to generate auxiliary data; however, the nature of these auxiliary data is not described.** The manuscript should clearly specify the auxiliary variables derived from the field measurements and explain their role in the analysis.
5. **Figure 2 presents a high-resolution Sentinel-2 image of Chengbu used for Random Forest classification.** However, the methodology for feature extraction from the Sentinel-2 imagery has not been described. The image preprocessing steps and feature extraction procedure should be included in the manuscript.
6. **The choice of the low-resolution Landsat dataset requires stronger justification, particularly since high-resolution Sentinel-2 imagery has also been used in the study.** Most of the analyses could potentially have been performed using Sentinel-2 data alone. The authors should explain why Landsat-derived indices (NDVI, EVI, NDWI, MNDWI, and SAVI) were preferred over the corresponding Sentinel-2-derived indices, despite the superior spatial resolution of Sentinel-2.
7. **The relationship among Fractional Vegetation Cover (FVC), time-series analysis, and satellite-derived data is not clearly explained.** The manuscript should elaborate on how these datasets and analytical components are integrated to develop the proposed generalized framework and contribute to the study outcomes.

8. **The selection of the Random Forest algorithm has not been adequately justified.** The authors should explain why Random Forest was chosen over other machine learning algorithms and discuss its suitability for the present application.
9. **Images of Figure 8 suggests that topographic factors have the least influence on FVC.** This observation requires further clarification and scientific justification to support the interpretation of the results.
10. **The rationale for employing the various analytical techniques presented in the study is not adequately explained.** A clear justification for each method, along with its specific role in the overall analytical framework, should be provided. Without such explanations, readers may find it difficult to understand the necessity and contribution of the individual techniques.

Overall Comment

The manuscript would benefit from a clearer explanation of the methodological framework, including the rationale behind each analytical component and the integration of the different datasets and techniques. Strengthening these aspects would improve the manuscript's clarity, coherence, and scientific contribution. There, major revision is suggested for possible publication in the journal.
