# Supplementary material for: Multi-feature fusion for grassland information extraction and temporal change monitoring
Source: PLoS One. 2026 Nov 6;21(11):e0347821. doi: 10.1371/journal.pone.0347821 (PMC13742955; doi:10.1371/journal.pone.0347821)
Supplement: Supplementary file 2 [file pone.0347821.s001.docx]

**Supplementary Table S1：Confusion Matrix of Scheme 2**

|  | Building | Cropland | Water | Forest | Bare land | Grass |
| --- | --- | --- | --- | --- | --- | --- |
| Building | 28078 | 20789 | 274 | 8992 | 0 | 357 |
| Cropland | 4713 | 60309 | 332 | 84986 | 0 | 1788 |
| Water | 113 | 370 | 6166 | 1664 | 0 | 73 |
| Forest | 1079 | 3910 | 78 | 1600916 | 1 | 417 |
| Bare land | 52 | 141 | 0 | 1103 | 450 | 83 |
| Grass | 85 | 288 | 0 | 3997 | 0 | 37275 |

The confusion matrix provides detailed information on the classification performance of Scheme 2, including the distribution of correctly and incorrectly classified samples among different land-cover categories. This result is provided to further evaluate the class-specific classification performance of the single-feature-based scheme**.**

**Supplementary Table S2：Confusion Matrix of Scheme 20**

|  | Building | Cropland | Water | Forest | Bare land | Grass |
| --- | --- | --- | --- | --- | --- | --- |
| Building | 33161 | 16184 | 198 | 8594 | 0 | 353 |
| Cropland | 4937 | 73242 | 307 | 72665 | 0 | 977 |
| Water | 143 | 490 | 6312 | 1365 | 0 | 76 |
| Forest | 1222 | 4128 | 71 | 1600436 | 1 | 543 |
| Bare land | 76 | 95 | 0 | 1143 | 504 | 11 |
| Grass | 84 | 191 | 0 | 2224 | 0 | 39146 |

The confusion matrix presents the detailed classification performance of the optimal multi-source feature fusion scheme (Scheme 20). Compared with Scheme 2, this result provides additional evidence for evaluating the effectiveness of integrating spectral, texture, topographic, and SAR features in improving grassland classification.

**Supplementary Figure S1:** OOB error curve of RF model

The figure illustrates the relationship between the number of decision trees and OOB accuracy during Random Forest training. The OOB accuracy gradually increases and becomes stable when the number of trees reaches approximately 300, indicating the convergence and robustness of the RF model used in this study.
