# Supplementary material for: Multi-feature fusion for grassland information extraction and temporal change monitoring
Source: PLoS One. 2026 Nov 6;21(11):e0347821. doi: 10.1371/journal.pone.0347821 (PMC13742955; doi:10.1371/journal.pone.0347821)
Supplement: Peer review history

# Peer review: Decision Letter 0

**DOI:** 10.1371/journal.pone.0347821.r001

**Authors:** Claudionor Silva

3 Aug 2026

PLOS One

Dear Dr. Kang,

Thank you for submitting your manuscript to PLOS ONE. After careful consideration, we feel that it has merit but does not fully meet PLOS ONE’s publication criteria as it currently stands. Therefore, we invite you to submit a revised version of the manuscript that addresses the points raised during the review process.

The current version of the manuscript requires substantial improvements before it can be considered for publication. The manuscript must be corrected in all points indicated by the reviewers. The main concerns are:

1) Lack of independent temporal validation for long-term mapping

2) Potential inconsistency among Landsat sensors is not addressed

3) Improvement achieved by feature fusion is relatively small and statistical significance is not evaluated

4) Absence of comparison with state-of-the-art methods (e.g.: XGBoost, LightGBM, CNN-based classifiers, Temporal CNNs, Transformers, ...)

5) Correlation analysis is insufficient for identifying driving mechanisms. Correlation analysis alone cannot establish causal relationships and may be strongly affected by confounding variables and spatial autocorrelation.

6) Unexpected negative relationship between precipitation and FVC requires deeper investigation. The authors should investigate this relationship more thoroughly and discuss.

7) Sample design needs more detail, e.g. number of samples per class, spatial distribution of samples, strategy used to avoid spatial autocorrelation, class balance.

8) Interpretation of feature importance should be more cautious. Feature importance values derived from Random Forest may be influenced by predictor correlation.

9) Transferability of the proposed framework remains untested. The study claims that the proposed framework is transferable to complex terrain regions. However, the framework was evaluated in only a single county.

10) O manuscrito contém também inconsistências na numeração das combinações de características e nas descrições dos resultados.

11) The abstract does not clearly convey the motivation behind the study. It should be revised the research motivation, the novelty of the proposed methodology, its key findings, and the rationale for adopting the proposed approach.

12) The authors state that "there are numerous challenges under complex surface conditions"; however, only the issue of short-term analysis has been discussed as a limitation of previous studies.

13) The novelty of the proposed methodology is not clearly articulated.

14) Section 2.2 states that grassland survey data obtained from field measurements were used to generate auxiliary data; however, the nature of these auxiliary data is not described.

15) Figure 2 presents a high-resolution Sentinel-2 image of Chengbu used for Random Forest classification. However, the methodology for feature extraction from the Sentinel-2 imagery has not been described.

16) The choice of the low-resolution Landsat dataset requires stronger justification, particularly since high-resolution Sentinel-2 imagery has also been used in the study.

17) The relationship among Fractional Vegetation Cover (FVC), time-series analysis, and satellite-derived data is not clearly explained.

18) The selection of the Random Forest algorithm has not been adequately justified. The authors should explain why Random Forest was chosen over other machine learning algorithms.

19) Images of Figure 8 suggests that topographic factors have the least influence on FVC. This observation requires further clarification and scientific justification to support the interpretation of the results.

20) The rationale for employing the various analytical techniques presented in the study is not adequately explained.

Please submit your revised manuscript by Sep 17 2026 11:59PM. If you will need more time than this to complete your revisions, please reply to this message or contact the journal office at plosone@plos.org. When you're ready to submit your revision, log on to https://www.editorialmanager.com/pone/ and select the 'Submissions Needing Revision' folder to locate your manuscript file.

- A letter that responds to each point raised by the academic editor and reviewer(s). You should upload this letter as a separate file labeled 'Response to Reviewers'.
- A marked-up copy of your manuscript that highlights changes made to the original version. You should upload this as a separate file labeled 'Revised Manuscript with Track Changes'.
- An unmarked version of your revised paper without tracked changes. You should upload this as a separate file labeled 'Manuscript'.

If you would like to make changes to your financial disclosure, please include your updated statement in your cover letter. Guidelines for resubmitting your figure files are available below the reviewer comments at the end of this letter.

If applicable, we recommend that you deposit your laboratory protocols in protocols.io to enhance the reproducibility of your results. Protocols.io assigns your protocol its own identifier (DOI) so that it can be cited independently in the future. For instructions see: https://journals.plos.org/plosone/s/submission-guidelines#loc-laboratory-protocols. Additionally, PLOS ONE offers an option for publishing peer-reviewed Lab Protocol articles, which describe protocols hosted on protocols.io. Read more information on sharing protocols at https://plos.org/protocols?utm_medium=editorial-email&utm_source=authorletters&utm_campaign=protocols.

As the corresponding author, your ORCID iD is verified in the submission system and will appear in the published article. PLOS supports the use of ORCID, and we encourage all coauthors to register for an ORCID iD and use it as well. Please encourage your coauthors to verify their ORCID iD within the submission system before final acceptance, as unverified ORCID iDs will not appear in the published article. *Only*  the individual author can complete the verification step; PLOS staff *cannot*  verify ORCID iDs on behalf of authors.

We look forward to receiving your revised manuscript.

Kind regards,

Claudionor Ribeiro da Silva

Academic Editor

PLOS One

Journal requirements:

When submitting your revision, we need you to address these additional requirements.

1. Please ensure that your manuscript meets PLOS ONE's style requirements, including those for file naming. The PLOS ONE style templates can be found at

https://journals.plos.org/plosone/s/file?id=wjVg/PLOSOne_formatting_sample_main_body.pdf and

https://journals.plos.org/plosone/s/file?id=ba62/PLOSOne_formatting_sample_title_authors_affiliations.pdf

2. We note that you have declared the use of generative AI in the preparation of your manuscript. Please note that contributions by artificial intelligence (AI) tools and technologies to a study or to an article’s contents must be clearly reported in a dedicated section of the Methods, or in the Acknowledgements section for article types lacking a Methods section. This section should include the name(s) of any tools used, a description of how the authors used the tool(s) and evaluated the validity of the tool’s outputs, and a clear statement of which aspects of the study, article contents, data, or supporting files were affected/generated by AI tool usage (https://journals.plos.org/plosone/s/ethical-publishing-practice#loc-artificial-intelligence-tools-and-technologies).

3. Please note that PLOS One has specific guidelines on code sharing for submissions in which author-generated code underpins the findings in the manuscript. In these cases, we expect all author-generated code to be made available without restrictions upon publication of the work. Please review our guidelines at https://journals.plos.org/plosone/s/materials-and-software-sharing#loc-sharing-code and ensure that your code is shared in a way that follows best practice and facilitates reproducibility and reuse.

4. Thank you for stating the following financial disclosure:

“Funding: Hunan Provincial Grassland Ecological Comprehensive Monitoring Project; Hunan Forestry Science and Technology Research and Innovation (XLKY202334)”

Please state what role the funders took in the study.  If the funders had no role, please state: "The funders had no role in study design, data collection and analysis, decision to publish, or preparation of the manuscript."

If this statement is not correct you must amend it as needed.

Please include this amended Role of Funder statement in your cover letter; we will change the online submission form on your behalf.

5. PLOS requires an ORCID iD for the corresponding author in Editorial Manager on papers submitted after December 6th, 2016. Please ensure that you have an ORCID iD and that it is validated in Editorial Manager. To do this, go to ‘Update my Information’ (in the upper left-hand corner of the main menu), and click on the Fetch/Validate link next to the ORCID field. This will take you to the ORCID site and allow you to create a new iD or authenticate a pre-existing iD in Editorial Manager.

6. We note that Figures 3 and 7 in your submission contain [map/satellite] images which may be copyrighted. All PLOS content is published under the Creative Commons Attribution License (CC BY 4.0), which means that the manuscript, images, and Supporting Information files will be freely available online, and any third party is permitted to access, download, copy, distribute, and use these materials in any way, even commercially, with proper attribution. For these reasons, we cannot publish previously copyrighted maps or satellite images created using proprietary data, such as Google software (Google Maps, Street View, and Earth). For more information, see our copyright guidelines: http://journals.plos.org/plosone/s/licenses-and-copyright.

We require you to either (1) present written permission from the copyright holder to publish these figures specifically under the CC BY 4.0 license, or (2) remove the figures from your submission:

1. You may seek permission from the original copyright holder of Figures 3 and 7 to publish the content specifically under the CC BY 4.0 license.

We recommend that you contact the original copyright holder with the Content Permission Form (http://journals.plos.org/plosone/s/file?id=7c09/content-permission-form.pdf) and the following text:

“I request permission for the open-access journal PLOS ONE to publish XXX under the Creative Commons Attribution License (CCAL) CC BY 4.0 (http://creativecommons.org/licenses/by/4.0/). Please be aware that this license allows unrestricted use and distribution, even commercially, by third parties. Please reply and provide explicit written permission to publish XXX under a CC BY license and complete the attached form.”

Please upload the completed Content Permission Form or other proof of granted permissions as an "Other" file with your submission.

In the figure caption of the copyrighted figure, please include the following text: “Reprinted from [ref] under a CC BY license, with permission from [name of publisher], original copyright [original copyright year].”

2. If you are unable to obtain permission from the original copyright holder to publish these figures under the CC BY 4.0 license or if the copyright holder’s requirements are incompatible with the CC BY 4.0 license, please either i) remove the figure or ii) supply a replacement figure that complies with the CC BY 4.0 license. Please check copyright information on all replacement figures and update the figure caption with source information. If applicable, please specify in the figure caption text when a figure is similar but not identical to the original image and is therefore for illustrative purposes only.

The following resources for replacing copyrighted map figures may be helpful:

USGS National Map Viewer (public domain): http://viewer.nationalmap.gov/viewer/

The Gateway to Astronaut Photography of Earth (public domain): http://eol.jsc.nasa.gov/sseop/clickmap/

Maps at the CIA (public domain): https://www.cia.gov/library/publications/the-world-factbook/index.html and https://www.cia.gov/library/publications/cia-maps-publications/index.html

NASA Earth Observatory (public domain): http://earthobservatory.nasa.gov/

Landsat: http://landsat.visibleearth.nasa.gov/

USGS EROS (Earth Resources Observatory and Science (EROS) Center) (public domain): http://eros.usgs.gov/#

Natural Earth (public domain): http://www.naturalearthdata.com/

If the reviewer comments include a recommendation to cite specific previously published works, please review and evaluate these publications to determine whether they are relevant and should be cited. There is no requirement to cite these works unless the editor has indicated otherwise.

[Note: HTML markup is below. Please do not edit.]

Reviewers' comments:

Reviewer's Responses to Questions

**Comments to the Author**

1. Is the manuscript technically sound, and do the data support the conclusions?

Reviewer #1: Partly

Reviewer #2: Partly

Reviewer #3: Yes

**********

2. Has the statistical analysis been performed appropriately and rigorously?

Reviewer #1: No

Reviewer #2: No

Reviewer #3: Yes

**********

3. Have the authors made all data underlying the findings in their manuscript fully available?

The PLOS Data policy

Reviewer #1: Yes

Reviewer #2: No

Reviewer #3: No

**********

4. Is the manuscript presented in an intelligible fashion and written in standard English?

Reviewer #1: Yes

Reviewer #2: Yes

Reviewer #3: Yes

**********

Reviewer #1: 1. Lack of independent temporal validation for long-term mapping

The manuscript uses training samples collected during 2021–2024 and applies a model trained using the 2025 dataset to reconstruct grassland maps for the entire 2000–2025 period. However, no independent validation data are provided for historical years.

Consequently, the reliability of the reconstructed grassland maps prior to the sample collection period remains unclear. The authors should justify the assumption of temporal transferability and, if possible, provide independent validation using historical imagery, existing land-cover products, or field records from earlier years.

2. Potential inconsistency among Landsat sensors is not addressed

The study combines Landsat 5, Landsat 8 and Landsat 9 observations over a 25-year period. These sensors differ in spectral response functions and radiometric characteristics.

The manuscript does not discuss whether inter-sensor harmonization was performed or how potential sensor-induced discontinuities may affect the observed grassland dynamics and FVC trends.

The authors should explicitly describe the harmonization procedure and evaluate whether detected temporal changes could partially result from sensor transitions rather than real ecological change.

3. Improvement achieved by feature fusion is relatively small and statistical significance is not evaluated

The proposed multi-feature fusion framework is presented as a major methodological contribution. However, the increase in OA between the best simpler models and the full-feature model is relatively limited (e.g., approximately 93.4–93.7% vs. 93.8%).

No statistical test is provided to demonstrate whether these improvements are significant.

4. Absence of comparison with state-of-the-art methods

The study only evaluates Random Forest under different feature combinations.

Recent grassland mapping studies increasingly employ advanced approaches such as XGBoost, LightGBM, CNN-based classifiers, Temporal CNNs, Transformers, or foundation-model-based representations.

5. Correlation analysis is insufficient for identifying driving mechanisms

The manuscript repeatedly refers to "driving mechanisms" of FVC variation, yet the analysis relies primarily on Pearson and Spearman correlations.

Correlation analysis alone cannot establish causal relationships and may be strongly affected by confounding variables and spatial autocorrelation.

The authors should either:

* moderate their interpretation and avoid causal language, or

* employ more appropriate methods such as partial correlation, multiple regression, geographically weighted regression (GWR), structural equation modeling (SEM), or machine-learning-based importance analysis.

6. Unexpected negative relationship between precipitation and FVC requires deeper investigation

One of the most surprising findings is the significant negative correlation between precipitation and FVC (r = –0.589).

This result contradicts the dominant ecological understanding for most grassland systems, where precipitation generally promotes vegetation growth.

The manuscript currently provides only a brief explanation. The authors should investigate this relationship more thoroughly and discuss:

* possible lag effects,

* collinearity with temperature and elevation,

* excessive rainfall impacts,

* data resolution mismatches,

* methodological artefacts.

7. Sample design needs more detail

The manuscript should report:

* number of samples per class,

* spatial distribution of samples,

* strategy used to avoid spatial autocorrelation,

* class balance.

Without this information the robustness of the classification assessment cannot be fully evaluated.

8. Interpretation of feature importance should be more cautious

Feature importance values derived from Random Forest may be influenced by predictor correlation.

Therefore, the ranking of NDVI, DEM, and other variables should not be interpreted as direct evidence of ecological importance.

The authors should discuss the limitations of permutation-based importance measures.

9. Transferability of the proposed framework remains untested

The study claims that the proposed framework is transferable to complex terrain regions.

However, the framework was evaluated in only a single county.

The authors should either:

* reduce the strength of this claim, or

* demonstrate transferability through testing in an independent region.

Reviewer #2: The study mainly focuses on grassland information extraction and temporal change monitoring in complex mountainous regions. The topic has certain applied value. However, the manuscript still has several key issues in methodological design, validation strategy, and reproducibility, which affect the reliability of the main conclusions.

1. The authors used samples from 2025 to train the Random Forest model and then applied it to classification and change analysis. However, independent validation for historical years is lacking, making it difficult to fully demonstrate the reliability of the long-term classification results.

2. The feature sets used in different time periods are inconsistent. The authors need to evaluate how this difference may affect the comparability of grassland area changes over time.

3. The description of sample size, spatial distribution, and related information is insufficient.

4. The manuscript also contains inconsistencies in feature-combination numbering and result descriptions, and the correlation analysis is further interpreted as evidence of driving mechanisms.

In summary, the manuscript requires substantial methodological restructuring and additional validation before it can be reconsidered. Therefore, I recommend rejecting this manuscript, while encouraging the authors to resubmit after sufficiently supplementing the validation and restructuring the analysis.

Reviewer #3: Review of the Article submitted in PLOS One

Article Title: Multi-Feature Fusion for Grassland Information Extraction and Temporal Change Monitoring

This study proposes a multi-feature fusion framework integrating optical, SAR, vegetation, texture, topographic, and time-series data within the Google Earth Engine platform, coupled with a Random Forest model, to improve grassland mapping accuracy in complex mountainous terrain. It further investigates the spatial and temporal dynamics of grasslands and identifies the relative influence of topographic, climatic, and anthropogenic factors on vegetation cover.

Article seems interesting. But it requires major revision for possible publication in the journal. The comments are as follows:

1. The abstract does not clearly convey the motivation behind the study. It should be revised to concisely highlight the research motivation, the novelty of the proposed methodology, its key findings, and the rationale for adopting the proposed approach.

2. The authors state that "there are numerous challenges under complex surface conditions"; however, only the issue of short-term analysis has been discussed as a limitation of previous studies. The other challenges should be clearly identified, explained, and supported with appropriate justification.

3. The novelty of the proposed methodology is not clearly articulated. The specific methodological contribution and its significance should be explicitly stated at the end of the Introduction.

4. Section 2.2 states that grassland survey data obtained from field measurements were used to generate auxiliary data; however, the nature of these auxiliary data is not described. The manuscript should clearly specify the auxiliary variables derived from the field measurements and explain their role in the analysis.

5. Figure 2 presents a high-resolution Sentinel-2 image of Chengbu used for Random Forest classification. However, the methodology for feature extraction from the Sentinel-2 imagery has not been described. The image preprocessing steps and feature extraction procedure should be included in the manuscript.

6. The choice of the low-resolution Landsat dataset requires stronger justification, particularly since high-resolution Sentinel-2 imagery has also been used in the study. Most of the analyses could potentially have been performed using Sentinel-2 data alone. The authors should explain why Landsat-derived indices (NDVI, EVI, NDWI, MNDWI, and SAVI) were preferred over the corresponding Sentinel-2-derived indices, despite the superior spatial resolution of Sentinel-2.

7. The relationship among Fractional Vegetation Cover (FVC), time-series analysis, and satellite-derived data is not clearly explained. The manuscript should elaborate on how these datasets and analytical components are integrated to develop the proposed generalized framework and contribute to the study outcomes.

8. The selection of the Random Forest algorithm has not been adequately justified. The authors should explain why Random Forest was chosen over other machine learning algorithms and discuss its suitability for the present application.

9. Images of Figure 8 suggests that topographic factors have the least influence on FVC. This observation requires further clarification and scientific justification to support the interpretation of the results.

10. The rationale for employing the various analytical techniques presented in the study is not adequately explained. A clear justification for each method, along with its specific role in the overall analytical framework, should be provided. Without such explanations, readers may find it difficult to understand the necessity and contribution of the individual techniques.

Overall Comment

The manuscript would benefit from a clearer explanation of the methodological framework, including the rationale behind each analytical component and the integration of the different datasets and techniques. Strengthening these aspects would improve the manuscript's clarity, coherence, and scientific contribution. There, major revision is suggested for possible publication in the journal.

**********

what does this mean?). If published, this will include your full peer review and any attached files.

If you choose “no”, your identity will remain anonymous but your review may still be made public.

**Do you want your identity to be public for this peer review?**  For information about this choice, including consent withdrawal, please see our Privacy Policy

Reviewer #1: No

Reviewer #2: No

Reviewer #3: **Yes:**  Dr. Ankur Biswas, ICAR-Indian Agricultural Statistics Research Institute (ICAR-IASRI), New Delhi

**********

[NOTE: If reviewer comments were submitted as an attachment file, they will be attached to this email and accessible via the submission site. Please log into your account, locate the manuscript record, and check for the action link "View Attachments". If this link does not appear, there are no attachment files.]

To ensure your figures meet our technical requirements, please review our figure guidelines: https://journals.plos.org/plosone/s/figures

You may also use PLOS’s free figure tool, NAAS, to help you prepare publication quality figures: https://journals.plos.org/plosone/s/figures#loc-tools-for-figure-preparation.

NAAS will assess whether your figures meet our technical requirements by comparing each figure against our figure specifications.

Attachment Submitted filename: Review comments_15.07.2026.pdf

# Author comment: Author response to Decision Letter 1

**DOI:** 10.1371/journal.pone.0347821.r002

13 Sep 2026

Reviewer #1:

We sincerely thank Reviewer #1 for the careful and constructive evaluation of our manuscript. The comments regarding temporal validation, Landsat sensor consistency, statistical significance of feature fusion, comparison with state-of-the-art methods, and the interpretation of correlation results are highly valuable. These suggestions have helped us identify important weaknesses and have guided substantial improvements in the revised manuscript. We are grateful for the reviewer’s time and expertise.

1. Lack of independent temporal validation for long-term mapping

The manuscript uses training samples collected during 2021–2024 and applies a model trained using the 2025 dataset to reconstruct grassland maps for the entire 2000–2025 period. However, no independent validation data are provided for historical years.

Consequently, the reliability of the reconstructed grassland maps prior to the sample collection period remains unclear. The authors should justify the assumption of temporal transferability and, if possible, provide independent validation using historical imagery, existing land-cover products, or field records from earlier years.

Response: We fully agree that independent historical validation is important. In the revised manuscript, we no longer claim that the historical classifications are fully validated. Section 4.3 now explicitly states that the 2000–2011 classifications were obtained through temporal transfer of the model trained with recent reference samples, and that independent validation for this period was not conducted because of the lack of historical field observations. The results for this period are therefore described as indicative representations of historical land-cover patterns rather than fully validated products. Section 4.5 further acknowledges the temporal transferability assumption and associated uncertainties. The Abstract has also been revised to avoid implying annual or fully validated long-term monitoring.

Changes made: Section 4.3, Section 4.5, Abstract.

2. Potential inconsistency among Landsat sensors is not addressed

The study combines Landsat 5, Landsat 8 and Landsat 9 observations over a 25-year period. These sensors differ in spectral response functions and radiometric characteristics.

The manuscript does not discuss whether inter-sensor harmonization was performed or how potential sensor-induced discontinuities may affect the observed grassland dynamics and FVC trends.

The authors should explicitly describe the harmonization procedure and evaluate whether detected temporal changes could partially result from sensor transitions rather than real ecological change.

Response: We agree that Landsat 5, Landsat 8, and Landsat 9 differ in spectral response and radiometric characteristics. In Section 2.2.1, we have clarified that all Landsat datasets were Collection 2 Level-2 products with consistent atmospheric correction, cloud masking, median compositing, spatial clipping, and resampling to 30 m. All datasets were referenced to WGS 1984. For FVC estimation, the 5th and 95th percentiles of annual NDVI distributions were determined separately for each year to reduce interannual and sensor-related biases. We acknowledge that formal cross-sensor harmonization was not performed. This limitation is now discussed in Section 4.5, and future cross-sensor calibration is proposed.

Changes made: Section 2.2.1, Section 3.6, Section 4.5.

3. Improvement achieved by feature fusion is relatively small and statistical significance is not evaluated

The proposed multi-feature fusion framework is presented as a major methodological contribution. However, the increase in OA between the best simpler models and the full-feature model is relatively limited (e.g., approximately 93.4–93.7% vs. 93.8%).

No statistical test is provided to demonstrate whether these improvements are significant.

Response: We agree that the differences among some high-performing schemes are small. In the revised manuscript, we have moderated the interpretation of Scheme 20 as the “best” scheme. Section 4.1 now states that the difference between Scheme 20 (OA = 93.79%, Kappa = 0.7165) and Scheme 16 (OA = 93.76%, Kappa = 0.7154) is very small and may partly reflect sampling variability. We also added grassland-specific F1-scores in Table 5 to provide a more balanced evaluation. We acknowledge that McNemar’s test or bootstrap confidence intervals were not applied; this is now identified as a future improvement.

Changes made: Section 4.1, Table 5, Section 4.5.

4. Absence of comparison with state-of-the-art methods

The study only evaluates Random Forest under different feature combinations.

Recent grassland mapping studies increasingly employ advanced approaches such as XGBoost, LightGBM, CNN-based classifiers, Temporal CNNs, Transformers, or foundation-model-based representations.

Response: We appreciate this comment. The primary objective of this study was not to propose a new classifier or to rank classifiers, but to systematically evaluate multi-source feature fusion for grassland extraction under complex mountainous conditions. Random Forest was selected because it is robust for high-dimensional heterogeneous remote sensing data, relatively tolerant to feature redundancy, and provides OOB permutation importance for feature contribution analysis. This rationale is now clearly stated in Section 3.4. We acknowledge that XGBoost, LightGBM, CNN, Temporal CNN, Transformer, and foundation-model-based approaches may further improve performance. This is now discussed as future work in Section 4.5.

Changes made: Section 3.4, Section 4.5.

5. Correlation analysis is insufficient for identifying driving mechanisms

The manuscript repeatedly refers to "driving mechanisms" of FVC variation, yet the analysis relies primarily on Pearson and Spearman correlations.

Correlation analysis alone cannot establish causal relationships and may be strongly affected by confounding variables and spatial autocorrelation.

The authors should either:

* moderate their interpretation and avoid causal language, or

* employ more appropriate methods such as partial correlation, multiple regression, geographically weighted regression (GWR), structural equation modeling (SEM), or machine-learning-based importance analysis.

Response: We agree. In the revised manuscript, we have carefully moderated causal language. Section 3.7 clarifies that Pearson and Spearman analyses identify statistical associations rather than causal mechanisms. Section 4.4 no longer describes the results as definitive driving mechanisms. Section 4.5 states that future studies should use partial correlation, multiple regression, geographically weighted regression (GWR), structural equation modeling (SEM), or interpretable machine learning methods such as SHAP to better quantify interactions and relative contributions.

Changes made: Section 3.7, Section 4.4, Section 4.5, Abstract, Conclusions.

6. Unexpected negative relationship between precipitation and FVC requires deeper investigation

One of the most surprising findings is the significant negative correlation between precipitation and FVC (r = –0.589).

This result contradicts the dominant ecological understanding for most grassland systems, where precipitation generally promotes vegetation growth.

The manuscript currently provides only a brief explanation. The authors should investigate this relationship more thoroughly and discuss:

* possible lag effects,

* collinearity with temperature and elevation,

* excessive rainfall impacts,

* data resolution mismatches,

* methodological artefacts.

Response: We agree that the negative FVC–precipitation relationship requires cautious interpretation. In the revised Section 4.4, we no longer interpret it as evidence that precipitation inhibits vegetation growth. Instead, we discuss it as a statistical association in a humid subtropical mountainous pasture system, where vegetation dynamics are jointly affected by radiation, cloud cover, soil moisture, temperature, management, and grazing. We also acknowledge that lag effects and collinearity with temperature/elevation were not quantitatively separated. These are now identified as future research directions.

Changes made: Section 4.4, Section 4.5.

7. Sample design needs more detail

The manuscript should report:

* number of samples per class,

* spatial distribution of samples,

* strategy used to avoid spatial autocorrelation,

* class balance.

Without this information the robustness of the classification assessment cannot be fully evaluated.

Response: We have substantially expanded Section 2.2.2. The revised manuscript reports the total number of labeled pixels and the number for each class: 2,875,111 labeled pixels in total, including 8,996 built-up land, 234,044 cropland, 12,900 water body, 2,471,387 forest, 2,813 bare land, and 64,069 grassland pixels. The dataset was randomly divided into 65% training (1,868,822 pixels) and 35% validation (1,006,289 pixels). A non-balanced sampling strategy was used to preserve the actual class distribution. The interpretation was conducted by a single experienced interpreter following consistent criteria; this is acknowledged as a limitation in Section 4.5. A sample distribution map can also be provided as supplementary material if required.

Changes made: Section 2.2.2, Section 4.5.

8. Interpretation of feature importance should be more cautious

Feature importance values derived from Random Forest may be influenced by predictor correlation.

Therefore, the ranking of NDVI, DEM, and other variables should not be interpreted as direct evidence of ecological importance.

The authors should discuss the limitations of permutation-based importance measures.

Response: We agree. Section 3.3 now states that OOB permutation importance reflects the contribution of variables to model prediction rather than direct causal or ecological importance. Section 4.2 has also been revised to interpret NDVI, DEM, moisture indices, texture, and SAR features cautiously, noting that predictor correlation may influence importance rankings. The ranking is presented as model-based evidence, not as proof of ecological causality.

Changes made: Section 3.3, Section 4.2.

9. Transferability of the proposed framework remains untested

The study claims that the proposed framework is transferable to complex terrain regions.

However, the framework was evaluated in only a single county.

The authors should either:

* reduce the strength of this claim, or

* demonstrate transferability through testing in an independent region.

Response: We agree that the original claim was too broad. In the revised Abstract and Introduction, we have replaced “transferable framework” with wording such as “methodological reference” and “practical reference for similar subtropical mountainous grassland environments.” We explicitly state that the framework was evaluated in one county and that cross-regional validation remains future work.

Changes made: Abstract, Introduction, Section 4.5, Conclusions.

We are very grateful to Reviewer #1 for the thorough review and for providing detailed and actionable suggestions. The comments have significantly improved the scientific rigor, transparency, and clarity of our study. We have carefully addressed each point in the revised manuscript and hope that the changes meet the reviewer’s expectations.

Reviewer #2:

The study mainly focuses on grassland information extraction and temporal change monitoring in complex mountainous regions. The topic has certain applied value. However, the manuscript still has several key issues in methodological design, validation strategy, and reproducibility, which affect the reliability of the main conclusions.

We sincerely appreciate Reviewer #2 for the rigorous and detailed review of our manuscript. The comments on historical validation, consistency of feature sets across time periods, sample description, feature-combination numbering, and the interpretation of correlation analysis are extremely helpful. These observations have enabled us to correct important inconsistencies and to strengthen the methodological transparency of the study. We thank the reviewer for the valuable input.

1. The authors used samples from 2025 to train the Random Forest model and then applied it to classification and change analysis. However, independent validation for historical years is lacking, making it difficult to fully demonstrate the reliability of the long-term classification results.

Response: This concern is addressed in the same way as Reviewer #1, Comment 1. Section 4.3 now clearly states that historical classifications for 2000–2011 are unvalidated extrapolations based on temporal model transfer. The results are interpreted with appropriate uncertainty language. Section 4.5 discusses the need for historical reference data, archived high-resolution imagery, and multi-period field observations.

Changes made: Section 4.3, Section 4.5.

2. The feature sets used in different time periods are inconsistent. The authors need to evaluate how this difference may affect the comparability of grassland area changes over time.

Response: We agree that this affects comparability. The revised manuscript explicitly states that 2015–2025 classifications used the five-feature combination (including SAR), whereas 2000–2011 classifications used the four-feature combination because Sentinel-1 SAR observations were not available before 2015. This limitation is now acknowledged in Section 4.3 and Section 4.5. The long-term trend interpretation is therefore cautious and focuses on overall stability and interannual fluctuations rather than precise year-to-year SAR-dependent comparisons.

Changes made: Section 4.3, Section 4.5.

3. The description of sample size, spatial distribution, and related information is insufficient.

Response: This has been addressed in Section 2.2.2. We now report total samples, per-class samples, training/validation numbers, sampling strategy, and interpretation procedure. The spatial representativeness is described as being based on manual delineation across the entire study area, covering different land-cover types, terrain conditions, and spatial environments. The single-interpreter limitation is acknowledged.

Changes made: Section 2.2.2, Section 4.5.

4. The manuscript also contains inconsistencies in feature-combination numbering and result descriptions, and the correlation analysis is further interpreted as evidence of driving mechanisms.

Response: We have carefully rechecked all scheme definitions. Scheme 11 is now consistently defined as A+B+D (topographic + optical index + SAR features) in Table 2, Table 4, Table 5, Section 4.1, and the Conclusions. The incorrect A+B+C labels have been corrected. We have also checked Table 1/Table 2 cross-references. Regarding correlation analysis, causal language has been removed; the results are now described as statistical associations rather than driving mechanisms.

Changes made: Table 2, Table 4, Table 5, Section 4.1, Conclusions, Section 3.7, Section 4.4.

In summary, the manuscript requires substantial methodological restructuring and additional validation before it can be reconsidered. Therefore, I recommend rejecting this manuscript, while encouraging the authors to resubmit after sufficiently supplementing the validation and restructuring the analysis.

We sincerely thank Reviewer #2 for the critical and constructive comments, which have greatly helped us improve the reliability and reproducibility of this work. We have carefully revised the manuscript to address all the issues raised, including the clarification of historical validation limitations and the correction of scheme numbering. We hope the revised version now satisfies the reviewer’s concerns.

Reviewer #3:

Review of the Article submitted in PLOS One

Article Title: Multi-Feature Fusion for Grassland Information Extraction and Temporal Change Monitoring

This study proposes a multi-feature fusion framework integrating optical, SAR, vegetation, texture, topographic, and time-series data within the Google Earth Engine platform, coupled with a Random Forest model, to improve grassland mapping accuracy in complex mountainous terrain. It further investigates the spatial and temporal dynamics of grasslands and identifies the relative influence of topographic, climatic, and anthropogenic factors on vegetation cover.

We deeply appreciate Reviewer #3 for the comprehensive and insightful review. The suggestions regarding the abstract, research motivation, methodological novelty, auxiliary data description, Sentinel-2 usage, Random Forest justification, and the rationale of analytical techniques are highly constructive. These comments have helped us substantially improve the logical structure and scientific contribution of the manuscript. We are grateful for the reviewer’s careful reading and professional guidance.

Article seems interesting. But it requires major revision for possible publication in the journal. The comments are as follows:

1. The abstract does not clearly convey the motivation behind the study. It should be revised to concisely highlight the research motivation, the novelty of the proposed methodology, its key findings, and the rationale for adopting the proposed approach.

Response: The Abstract has been revised to include the research motivation, the multi-feature fusion framework, the GEE-based workflow, key accuracy metrics (OA = 93.79%, Kappa = 0.7165), long-term grassland area stability, FVC variability (mostly 0.59–0.72 in most years), localized degradation in southwestern Nanshan Pasture, and the main correlation findings. It also states that the identified relationships are statistical associations rather than causal mechanisms.

Changes made: Abstract.

2. The authors state that "there are numerous challenges under complex surface conditions"; however, only the issue of short-term analysis has been discussed as a limitation of previous studies. The other challenges should be clearly identified, explained, and supported with appropriate justification.

Response: The Introduction has been expanded to discuss complex terrain, elevation gradients, slope and illumination differences, fragmented grassland distribution, spectral confusion among grassland, forest, cropland, and shrub, and the limitations of single-source or short-term observations in subtropical mountainous regions.

Changes made: Introduction.

3. The novelty of the proposed methodology is not clearly articulated. The specific methodological contribution and its significance should be explicitly stated at the end of the Introduction.

Response: We have added a concise statement of objectives and contributions at the end of the Introduction. The contributions include: (1) a systematic 20-scheme multi-feature evaluation strategy for subtropical mountainous grassland; (2) a long-term monitoring framework for a fragmented mountainous grassland region; and (3) integration of FVC trend analysis with topographic, climatic, and anthropogenic factor analysis. We also clarify that the novelty lies in the integrated workflow rather than in a new classifier.

Changes made: Introduction.

4. Section 2.2 states that grassland survey data obtained from field measurements were used to generate auxiliary data; however, the nature of these auxiliary data is not described. The manuscript should clearly specify the auxiliary variables derived from the field measurements and explain their role in the analysis.

Response: Section 2.2.4 has been expanded. It now reports 102 field survey points collected during 2021–2024 (19 in 2021, 29 in 2022, 27 in 2023, and 27 in 2024). GNSS coordinates were recorded and spatially matched with remote sensing pixels. These field data were used for land-cover reference sample construction, not as independent validation samples. The human activity intensity proxy dataset is also described, including residential areas, major roads, Euclidean distance analysis, reclassification, and raster calculation.

Changes made: Section 2.2.4.

5. Figure 2 presents a high-resolution Sentinel-2 image of Chengbu used for Random Forest classification. However, the methodology for feature extraction from the Sentinel-2 imagery has not been described. The image preprocessing steps and feature extraction procedure should be included in the manuscript.

Response: We have clarified that Sentinel-2 imagery was not used as an input feature for Random Forest classification. It was used only to assist visual interpretation and reference sample delineation together with Google Earth high-resolution imagery and field survey data. The RF features were derived from Landsat, Sentinel-1 SAR, and SRTM DEM. Figure 2 has been corrected accordingly.

Changes made: Section 2.2.1, Section 2.2.2, Figure 2.

6. The choice of the low-resolution Landsat dataset requires stronger justification, particularly since high-resolution Sentinel-2 imagery has also been used in the study. Most of the analyses could potentially have been performed using Sentinel-2 data alone. The authors should explain why Landsat-derived indices (NDVI, EVI, NDWI, MNDWI, and SAVI) were preferred over the corresponding Sentinel-2-derived indices, despite the superior spatial resolution of Sentinel-2.

Response: Landsat was selected because it provides consistent 30 m observations from 2000 to 2025, which is essential for long-term monitoring. Sentinel-2 data are only available after 2015 and were therefore not suitable for the full study period. Sentinel-2 was used only for visual interpretation and sample construction. All datasets were resampled to 30 m to maintain spatial consistency. This rationale is now stated in Section 2.2.1.

Changes made: Section 2.2.1.

7. The relationship among Fractional Vegetation Cover (FVC), time-series analysis, and satellite-derived data is not clearly explained. The manuscript should elaborate on how these datasets and analytical components are integrated to develop the proposed generalized framework and contribute to the study outcomes.

Response: Section 3.6 has been expanded. It explains that grassland extraction provides the spatial mask, annual NDVI is used in the Pixel Dichotomy Model to estimate FVC, and slope-based trend analysis is applied to characterize vegetation condition changes. The 5th and 95th percentiles of annual NDVI are used as soil and vegetation endmembers. Grassland area and FVC are jointly analyzed to distinguish quantity changes from quality changes.

Changes made: Section 3.6.

8. The selection of the Random Forest algorithm has not been adequately justified. The authors should explain why Random Forest was chosen over other machine learning algorithms and discuss its suitability for the present application.

Response: Section 3.4 now explains that RF was selected because it handles high-dimensional, nonlinear, heterogeneous remote sensing data, is relatively robust to feature redundancy and multicollinearity, and provides OOB permutation importance for feature contribution analysis. The study objective was feature-fusion evaluation, not classifier benchmarking. Future comparison with XGBoost, LightGBM, and deep learning is proposed.

Changes made: Section 3.4, Section 4.5.

9. Images of Figure 8 suggests that topographic factors have the least influence on FVC. This observation requires further clarification and scientific justification to support the interpretation of the results.

Response: We have clarified this point. DEM shows a significant negative correlation with FVC, indicating the importance of elevation gradients. Slope and aspect show only very weak correlations, meaning micro-topographic factors have limited explanatory power. This may be related to pasture management and the presence of managed or artificial grasslands. The distinction between macro-topographic elevation and micro-topographic slope/aspect is now discussed in Section 4.4.

Changes made: Section 4.4, Table 6, Figure 8.

10. The rationale for employing the various analytical techniques presented in the study is not adequately explained. A clear justification for each method, along with its specific role in the overall analytical framework, should be provided. Without such explanations, readers may find it difficult to understand the necessity and contribution of the individual techniques.

Response: We have strengthened the methodological rationale. The 20 feature-combination schemes evaluate feature complementarity; RF provides classification and OOB feature importance; OA/Kappa/UA/PA/F1 provide multi-metric accuracy assessment; PDM estimates FVC; Mann–Kendall and Sen’s slope test temporal trends; Pearson and Spearman correlations identify statistical associations. These roles are now explained in Sections 3.3–3.7 and summarized in the overall workflow.

Changes made: Sections 3.1, 3.3, 3.4, 3.5, 3.6, 3.7.

Overall Comment

The manuscript would benefit from a clearer explanation of the methodological framework, including the rationale behind each analytical component and the integration of the different datasets and techniques. Strengthening these aspects would improve the manuscript's clarity, coherence, and scientific contribution. There, major revision is suggested for possible publication in the journal.

We would like to express our sincere gratitude to Reviewer #3 for the detailed and thoughtful suggestions. The comments have guided us to better articulate the research gap, methodological framework, and the integration of multi-source data and analytical components. We have thoroughly revised the manuscript accordingly and believe it is now much stronger as a result. We thank the reviewer again for the valuable time and effort.

Additional Editorial and Consistency Corrections

In addition to the point-by-point responses above, we have made the following corrections:

1. Scheme 11 is consistently defined as A+B+D throughout the manuscript.

2. Table 1 and Table 2 references have been checked and corrected.

3. The description of temporal sampling has been revised to avoid implying continuous annual monitoring. The study uses selected cloud-free years between 2000 and 2025.

4. The Abstract now reports OA and Kappa for the optimal scheme.

5. FVC reporting in the Abstract has been revised to emphasize the common range during most years rather than the single historical extreme.

6. Causal language has been removed and replaced with “statistical association” where appropriate.

7. The Limitations and Future Perspectives section has been expanded to cover historical validation, sensor harmonization, spatial autocorrelation, uncertainty quantification, FVC validation, and cross-regional transferability.

8. Figures and tables have been checked for readability, labeling, and consistency.

9. The manuscript has been carefully edited for English language, formatting, and citation consistency.

10. New references have been added to support the literature review, discussion, and future perspectives, and all citations have been checked for formatting consistency and publication status.

________________________________________

We sincerely thank the editor and reviewers again for their constructive comments. We believe that the revised manuscript is now more transparent about its assumptions, limitations, and uncertainty, while more clearly presenting its methodological contribution and ecological implications. We hope the revised version is now suitable for further consideration.

# Peer review: Decision Letter 1

**DOI:** 10.1371/journal.pone.0347821.r003

**Authors:** Claudionor Silva

2 Oct 2026

Multi-Feature Fusion for Grassland Information Extraction and Temporal Change Monitoring

PONE-D-26-17204R1

Dear Dr. Kang,

We’re pleased to inform you that your manuscript has been judged scientifically suitable for publication and will be formally accepted for publication once it meets all outstanding technical requirements.

Within one week, you’ll receive an e-mail detailing the required amendments. When these have been addressed, you’ll receive a formal acceptance letter and your manuscript will be scheduled for publication.

An invoice will be generated when your article is formally accepted. Please note, if your institution has a publishing partnership with PLOS and your article meets the relevant criteria, all or part of your publication costs will be covered. Please make sure your user information is up-to-date by logging into Editorial Manager at Editorial Manager® and clicking the ‘Update My Information' link at the top of the page. For questions related to billing, please contact billing support.

If your institution or institutions have a press office, please notify them about your upcoming paper to help maximize its impact. If they’ll be preparing press materials, please inform our press team as soon as possible -- no later than 48 hours after receiving the formal acceptance. Your manuscript will remain under strict press embargo until 2 pm Eastern Time on the date of publication. For more information, please contact onepress@plos.org.

Kind regards,

Claudionor Ribeiro da Silva

Academic Editor

PLOS One

Additional Editor Comments (optional):

Reviewers' comments:

Reviewer's Responses to Questions

**Comments to the Author**

Reviewer #1: All comments have been addressed

Reviewer #3: All comments have been addressed

**********

2. Is the manuscript technically sound, and do the data support the conclusions?

Reviewer #1: Partly

Reviewer #3: Yes

**********

3. Has the statistical analysis been performed appropriately and rigorously?

Reviewer #1: Yes

Reviewer #3: Yes

**********

4. Have the authors made all data underlying the findings in their manuscript fully available?

The PLOS Data policy

Reviewer #1: No

Reviewer #3: No

**********

5. Is the manuscript presented in an intelligible fashion and written in standard English?

Reviewer #1: Yes

Reviewer #3: Yes

**********

Reviewer #1: (No Response)

Reviewer #3: All comments have been addressed. Data availability statement may be given in the paper as per the journal's policy.

**********

what does this mean?). If published, this will include your full peer review and any attached files.

If you choose “no”, your identity will remain anonymous but your review may still be made public.

**Do you want your identity to be public for this peer review?**  For information about this choice, including consent withdrawal, please see our Privacy Policy

Reviewer #1: No

Reviewer #3: No

**********

# Editor report: Acceptance letter

**DOI:** 10.1371/journal.pone.0347821.r004

**Authors:** Claudionor Silva

PONE-D-26-17204R1

PLOS One

Dear Dr. Kang,

I'm pleased to inform you that your manuscript has been deemed suitable for publication in PLOS One. Congratulations! Your manuscript is now being handed over to our production team.

At this stage, our production department will prepare your paper for publication. This includes ensuring the following:

* All references, tables, and figures are properly cited

* All relevant supporting information is included in the manuscript submission,

* There are no issues that prevent the paper from being properly typeset

You will receive further instructions from the production team, including instructions on how to review your proof when it is ready. Please keep in mind that we are working through a large volume of accepted articles, so please give us a few days to review your paper and let you know the next and final steps.

Lastly, if your institution or institutions have a press office, please let them know about your upcoming paper now to help maximize its impact. If they'll be preparing press materials, please inform our press team within the next 48 hours. Your manuscript will remain under strict press embargo until 2 pm Eastern Time on the date of publication. For more information, please contact onepress@plos.org.

You will receive an invoice from PLOS for your publication fee after your manuscript has reached the completed accept phase. If you receive an email requesting payment before acceptance or for any other service, this may be a phishing scheme. Learn how to identify phishing emails and protect your accounts at https://explore.plos.org/phishing.

If we can help with anything else, please email us at customercare@plos.org.

Thank you for submitting your work to PLOS One and supporting open access.

Kind regards,

PLOS One Editorial Office Staff

on behalf of

Dr. Claudionor Ribeiro da Silva

Academic Editor

PLOS One
